# TAD conservation in vertebrate genomes is driven by stabilising selection

**DOI:** 10.1186/s12915-025-02362-0

**Published:** 2025-08-05

**Authors:** Fabiana Patalano, Simen Rød Sandve, Rein Aasland, Jonas Paulsen

**Affiliations:** 1 Department of Biosciences, University of Oslo, 0316 Oslo, Norway; 2 Department of Animal and Aquacultural Sciences, Faculty of Biosciences, Norwegian University of Life Sciences, 1432 Ås, Norway

**Keywords:** Topologically associating domains, TAD, Stabilising selection, Evolution, 3D genome architecture, Hi-C, OU model, Genomic regulatory blocks, Synteny

## Abstract

**Background:**

Topologically associating domains (TADs) are fundamental structural and gene regulatory components of chromatin defined by regions of high intra-domain contact frequency. Though TADs are found across diverse metazoans, the extent of their evolutionary conservation is still debated.

**Results:**

Here, we investigated the evolutionary conservation of TADs by analysing Hi-C data from 12 vertebrate species. We examined TAD numbers, borders, and gene positioning within TADs. We found that TAD features are all highly conserved across species, but decrease with evolutionary distance. Nevertheless, modelling TAD evolution using Ornstein–Uhlenbeck (OU) process revealed strong stabilising selection signatures for TAD number within the majority of syntenic blocks. These syntenic blocks under selection were enriched for highly conserved noncoding elements associated with developmental gene regulation (genomic regulatory blocks). However, strong signatures for stabilising selection for TAD numbers were also found independent of genomic regulatory blocks or genes with non-developmental functions.

**Conclusions:**

These findings improve our understanding of TAD conservation and highlight stabilising selection as an important driver in 3D genome evolution. Although selection on TAD structures is pronounced for developmental genes, our findings highlight the importance of TADs in genome and organismal functions beyond developmental biology.

**Supplementary Information:**

The online version contains supplementary material available at 10.1186/s12915-025-02362-0.

## Background

In eukaryotes, genomic DNA is tightly packed with histones and non-histone proteins to form chromatin, which serves to protect and regulate genetic material while efficiently organising the long DNA molecules within the limited space of the nucleus. This organisation is hierarchical, with structural and regulatory processes working together to ensure genome function and stability [1].

A key feature of this dynamic organisation is the formation of topologically associating domains (TADs), sub-megabase-scale chromatin segments characterised by high intra-domain contact frequencies [2–5]. TADs typically contain genes interacting with regulatory elements within the same domain [6]. Interactions across TADs are restricted by border elements enriched with insulator proteins, such as CCCTC-binding factor (CTCF). The proposed mechanism for TAD formation involves loop extrusion, where cohesin interacts with CTCF, halting when cohesin encounters convergently orientated CTCF-bound sites [2, 7–11].

While first discovered in mammals, TADs have since been observed in many species, from *Drosophila* to prokaryotes and in plants [4, 12–14]. Vertebrates rely on CTCF for TAD borders, while other species use alternative proteins, suggesting independent evolution of some TAD mechanisms [15].

Although significant progress has been made in understanding properties of TADs, the precise mechanisms underlying their evolutionary origins and conservation are not known. In particular, the degree of TAD evolutionary conservation is still debated. While some evidence supports a considerable TAD conservation [2, 16–18], recent direct comparisons of TAD borders have reported a low level of conservation, challenging this notion [19, 20].

Growing evidence, direct and indirect, suggests that TAD borders could play a key role as functional elements. Indirect evidence of TAD conservation comes from studies examining genomic synteny breakpoints and the occurrence of CTCF-binding sites across species. Recent studies have revealed that synteny breakpoints in various mammals [17, 18, 21] and flies [22] are concentrated at TAD borders and depleted within TAD bodies, suggesting TADs are conserved during evolution [17, 18]. The conservation of TADs has also been inferred through comparative analyses of CTCF-binding sites and insulator activity across species [16], although this study does not report the number or proportion of conserved TADs observed [16]. Direct evidence of TAD conservation comes from several studies that compare TAD borders across different species. Initially, TADs were directly compared between human and mouse revealing that approximately 54% of human TAD borders are conserved in mouse [2]. Border conservation was defined based on the presence or absence of borders across species. Using a similar approach with more stringent criteria, a comparison of human and chimpanzee TAD borders found that only 43% are shared between the two species [23]. In a more recent study applying the same presence/absence criterion, it was estimated that only 14% of human and 15% of mouse TAD borders are conserved across 8 primate and rodent species [20], challenging earlier reports of TAD border conservation. This approach relies on whole-genome alignment and liftover processes, which becomes progressively more difficult as evolutionary distance increases.

Hence, when comparing multiple distantly related species, approaches that do not rely on base-pair-level orthology is necessary. In this context, synteny becomes a useful framework. Synteny (meaning “same ribbon” as introduced by Renwick [24]) and refers to the preserved order of genes or genomic segments across species. As gene order is preserved across much larger evolutionary distances, syntenic blocks therefore represent a practical and evolutionary meaningful unit to explore patterns of TAD conservation.

Beyond gene order, some genomic regions exhibit strong structural and functional conservation, in particular some genomic regulatory blocks (GRBs)—large, evolutionarily constrained regions housing key developmental genes alongside highly conserved noncoding elements (CNEs) [25–27]. These GRBs are often interspersed with phylogenetically and functionally unrelated “bystander” genes, which are not specifically regulated by the CNEs and have a distinct expression pattern [27]. The conservation of GRBs within syntenic blocks suggests that selection acts not only to maintain individual genes and their regulatory elements but also to preserve the broader genomic architecture that enables their coordinated function. The organisation of GRBs closely aligns with TADs, which form chromatin loops through cohesin and CTCF interactions, insulating regulatory landscapes and maintaining proper gene expression [26, 28]. The overlap between GRBs, synteny, and TADs suggests that TADs may act as structural scaffolds, preserving cis-regulatory interactions and ensuring the proper regulation of developmental genes across evolutionary timescales.

In this study, we quantify the conservation of TADs in a broader spectrum of vertebrate species using several TAD characteristics, such as TAD number, borders, and gene positioning within TADs. We then use an Ornstein–Uhlenbeck process to model the evolution of these TAD characteristics, and show that TAD conservation is in many cases driven by stabilising selection.

## Results

### Identification of syntenic blocks across 12 species

**Fig. 1:**
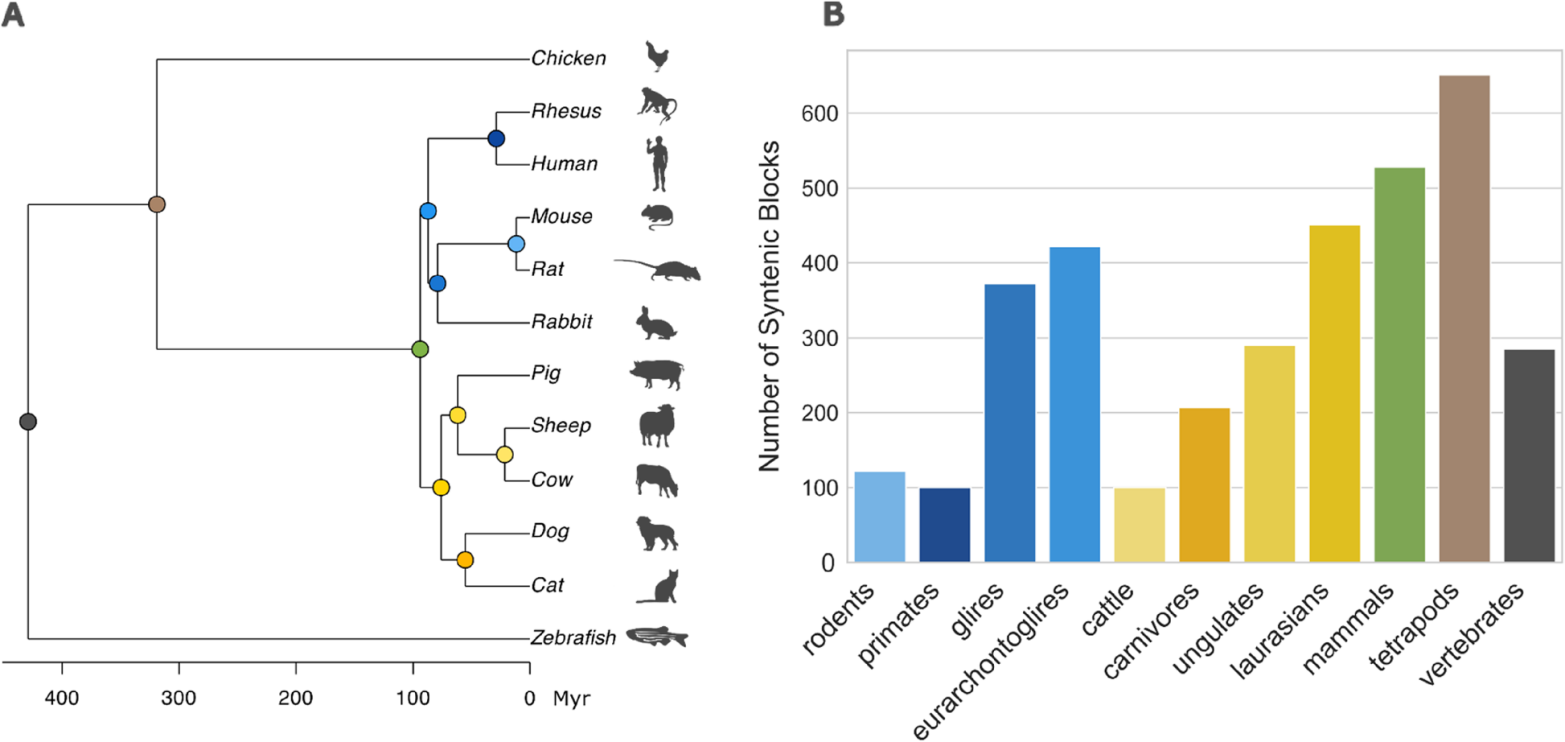
Phylogenetic distance affects the overall synteny conservation pattern across vertebrates. **A** Overview of the 12 vertebrate species included in the study. The estimated divergence times were obtained from http://timetree.org/. The figure was created using R, and the icons have been downloaded from https://www.phylopic.org/. **B** Bar plot illustrating the number of conserved syntenic blocks identified for each alignment. The minimum number of blocks observed is primates, while the maximum in tetrapods

To systematically explore TAD evolution, we compiled a dataset across the vertebrate lineage, including 12 species—10 mammalian species, chicken, and zebrafish (Fig. 1A). All species feature high-quality genome assemblies and available Hi-C data obtained from fibroblast cell lines. While this dataset provides valuable insight into TAD evolution within vertebrates, it is important to note that our coverage is limited, largely due to the availability of Hi-C data. Similar studies have used syntenic-block-based strategies to enhance comparative TAD analyses [2, 16, 23]. Following this approach, we analysed TADs within syntenic blocks to ensure meaningful cross-species comparisons while minimising alignment artefacts that increase with evolutionary distance. This method also allows for a more comprehensive investigation of TAD configurations by going beyond isolated TADs, specifically examining how TADs are organised in relation to each other, including their spatial arrangement.

For each ancestral node in the phylogenetic tree, we identified conserved syntenic blocks—genomic segments with preserved order of genes across species evolving from that node—using Cyntenator [29]. A synteny block consists of at least two orthologous genes across genomes. The number of syntenic blocks varies, ranging from 100 between humans and rhesus monkeys to 651 when including all mammals and chicken (Fig. 1B). As expected, the evolutionary distance between species impacts the distribution of gene counts per block; for example, the rodent alignment (13 million years divergence) shows up to 1767 genes per block (median ± SD: 31 ± 369), while alignments across all species (429 million years divergence) reveal a maximum of only 16 genes (median ± SD: 3 ± 2.27). Closely related species exhibit narrower gene count distributions, with an interquartile range of 144 for rodents compared to just 2 for the vertebrate alignment. Alignment coverage decreases with evolutionary distance, from over 90% to under 20% (additional details can be found in Additional file 2: Table S1). The reduction of 4194 genes within syntenic blocks when going from tetrapods to vertebrates (Additional file 2: Table S2) may result from the additional whole-genome duplication in teleost fish ~ 200 million years ago, followed by selective gene retention or loss (31). Analysis of these “missing” genes reveals that 34% (1425 genes) exhibit over 70% identity with non-syntenic paralogs, suggesting their relocation to non-syntenic regions rather than their complete absence from the zebrafish genome. Nevertheless, the percentage drops to 15% for genes sharing over 80% identity.

Gene Ontology (GO) analysis of genes conserved across all 12 species showed significant (*p* value < 0.05) enrichment in processes crucial for development and cellular regulation. Key enriched terms include organ development and anterior–posterior pattern specification (Additional file 1: Fig. S1).

### Identification of TADs in the vertebrate fibroblast genomes

We then performed 3D genome analysis on publicly available Hi-C data (GEO: GSE167581, SRA IDs: PRJNA482496, [30, 31]) of fibroblast cells. Our study included 12 Hi-C libraries, which we merged after iterative correction and KR normalisation.

This process resulted in an average of 295 million valid reads per species, with the human dataset containing the highest number of valid reads (568 million), while the zebrafish dataset had the lowest (36.6 million) (Additional file 1: Fig. S2).

Using HiCExplorer [32], we identified an average of 3278 TADs across the 12 vertebrate genomes (mean ± SD: 3.28 ± 818). The identified TADs ranged from 1207 in zebrafish to 4037 in rhesus (Additional file 1: Fig. S3A). This variation in TAD numbers across species may be attributed to differences in Hi-C resolution. The presence of TADs within syntenic blocks differed greatly between species, as expected, since blocks cover less of the genome, with the highest inclusion rate of 99% observed in the common ancestor of humans and rhesus macaques and the lowest rate of 12% between humans and zebrafish (Additional file 2: Table S3, Additional file 1: Fig. S3B).

### TAD number is conserved across vertebrate evolution

We analysed the rate of evolutionary change in TAD features across species, comparing pairs of species without using human as a reference. This approach allowed us to assess TAD conservation independently across species with similar divergence times, such as mouse, cow, and dog, which diverged approximately 70–100 million years ago.

**Fig. 2:**
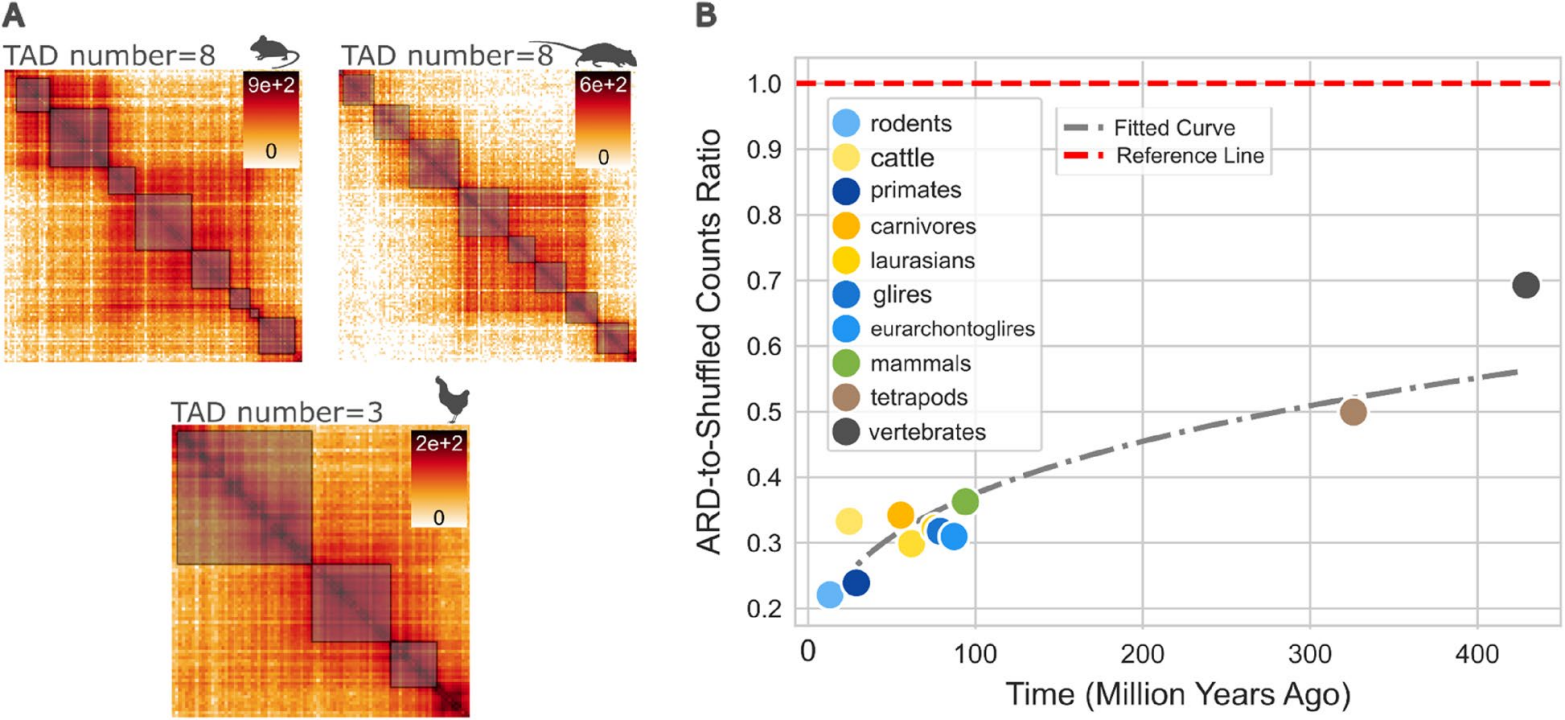
The TAD number is conserved, and this conservation correlates with evolutionary divergence. **A** Hi-C map illustrating TAD distributions within a syntenic block shared among mouse, rat, and chicken genomes (rat: chr9 61,755,803–66120277; mouse: chr1 74,793,498–74,938,002; chicken: chr7 21,976,097–2202637). The heatmap shows identical TAD numbers within syntenic blocks of mouse and rat, while a lower number of TADs is observed in the chicken syntenic blocks. **B** Scatterplot showing TAD number conservation is significantly higher than expected by chance (*p* value < 0.05). The observed conservation ranges from 0.22 to 0.69-fold lower than a random distribution

Analyses of evolutionary conservation of TAD numbers within syntenic blocks revealed an average change of 0.018 TADs per megabase pair (Mbp) per million years (Mbp/Myr) (Additional file 1: Fig. S4). Note that these change estimates assume a somewhat unrealistic linear relationship [33], but nevertheless provide an indication of the extent of TAD change over time. TAD number conservation decreases as evolutionary distance between species increases. This is exemplified also in Fig. 2A, where species sharing a more recent common ancestor, such as the mouse and rat, show an identical count of TADs within syntenic blocks in contrast to the more distantly related chicken, which does not.

To assess conservation and statistical significance across all alignments, we computed the average relative difference (ARD), which measures the proportional mean difference in TAD numbers, with lower values indicating stronger conservation. This provides insights into the stability of TAD numbers and helps evaluate conservation across varying evolutionary distances. To evaluate statistical significance, we compared our scores to a random distribution generated by shuffling the TAD counts within the syntenic blocks 1000 times. This method provides a baseline to determine if the observed conservation is significantly greater than what would be expected by chance.

Our findings revealed that TAD number conservation is significantly higher than would be expected by chance (*p* value < 0.05, Additional file 1: Fig. S5). The conservation we observed ranged from 0.22 to 0.69-fold less than the random distribution (Additional file 1: Fig. S2B). Taking primates as an example, the TAD conservation was nearly 0.2 times lower than the random. This pattern might suggest a strong phylogenetic signal, reflecting a non-random distribution of TAD number among these species.

### Divergence time correlates with TAD border position conservation in vertebrates

To determine if TAD border positions are evolutionarily conserved within syntenic blocks, we selected blocks with an equal number of TADs, averaging 40 blocks per species pair comparison.

**Fig. 3:**
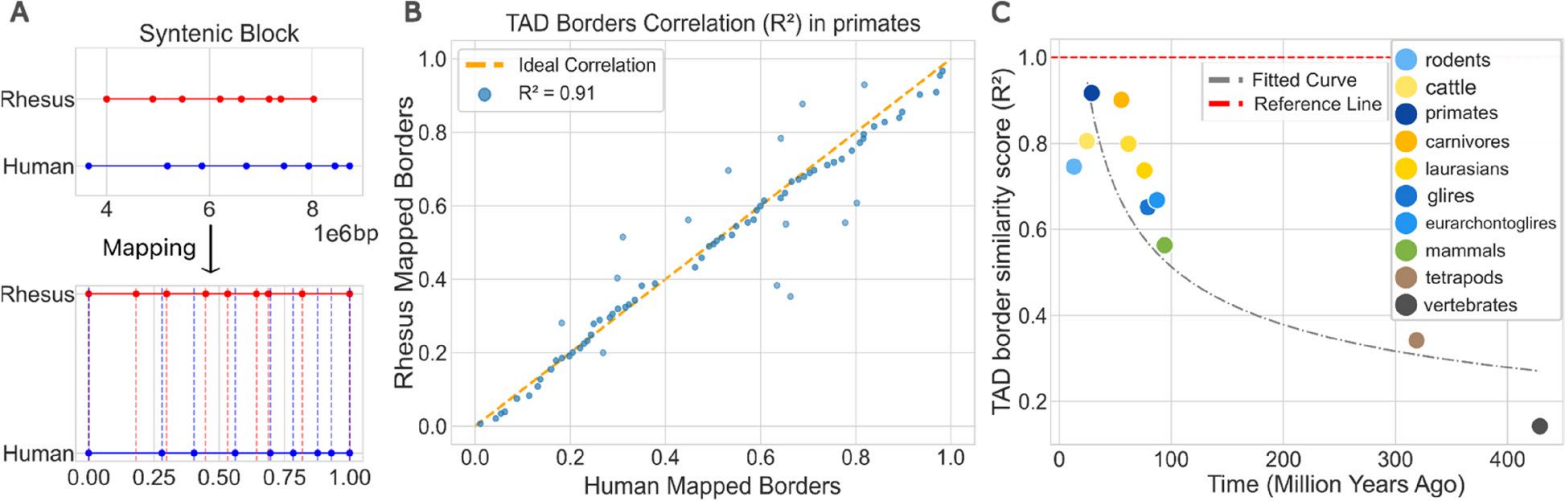
TAD borders are evolutionary stable in syntenic blocks across the 12 species. **A** Diagram illustrating TAD borders in a syntenic block. The top panel maps borders in rhesus and human along real genomic coordinates (x-axis in bp). The bottom panel normalises these borders to a 0–1 range while preserving relative distances. Dashed lines indicate where TAD borders align between species. **B** Scatter plot showing all the TAD borders of syntenic blocks with the same number of TADs along with the calculated *R*^2^ value of 0.91. **C** Scatter plot showing the average *R*^2^ scores for each clade, where higher *R*^2^ values indicate greater similarity and conservation of TAD border locations, and lower *R*^2^ values indicate greater divergence in border positions

Avoiding liftover, which loses reliability with increasing evolutionary distance, we normalised TAD border positions to a 0 to 1 scale within each syntenic block (Fig. 3A). We computed TAD border position conservation using the mean absolute differences in these relative border locations across all pairs of species. Dividing the species into three distinct clades (mammals, tetrapods, and vertebrates) revealed an average difference in border position of ~ 9.2% within mammals, 10.7% in tetrapods, and ~ 14.2% in vertebrates (Additional file 1: Fig. S6). To exemplify this, we assessed mean absolute differences in the *SIX2/3* cluster, a region known to be TAD-regulated in zebrafish (27). In this cluster, all the border positions are conserved across different species pairs, and the absolute difference between pairs consistently stays under 0.2 (Additional file 1: Fig. S7), providing confidence to this metric.

To further compare relative TAD border positions between species pairs, we calculated the *R*^2^ score, which measures the proportion of variance in the border positions of one species that can be explained by the positions in another species (Fig. 3A and B). Applying this analysis to the entire dataset, we find that relative border positions have a statistically significant correlation for all clades (Additional file 2: Table S4), yet with a gradually decreasing *R*^2^ values over evolutionary time (Fig. 3C). Additionally, we analysed the border outliers in primate alignments and found that, even though they are not statistically enriched in any GO term, these genes are involved in processes such as glucose response and immune response (Additional file 1: Fig. S8).

### Gene position is conserved within TADs

Because TADs serve to restrict gene regulatory interactions [6], we next investigated whether genes tend to stay within the same TADs over evolutionary time. To quantify this, we computed the average number of genes found within the same TAD across pairs of species within syntenic blocks. This revealed that mammalian synteny blocks have an average of 9.7 orthologous genes per Mbp/Myr. Given the average density of ~ 14 genes per Mbp in these blocks overall, this indicates a relatively high degree of gene content conservation within TADs. Similarly, for tetrapods, we observe a conservation of 9.3 genes per Mbp/Myr, yet for the whole vertebrate clade the conservation score drops to about 5 genes per Mbp per million years, reflecting a more substantial divergence in gene positional conservation as the evolutionary distance increases (Additional file 1: Fig. S9).

**Fig. 4:**
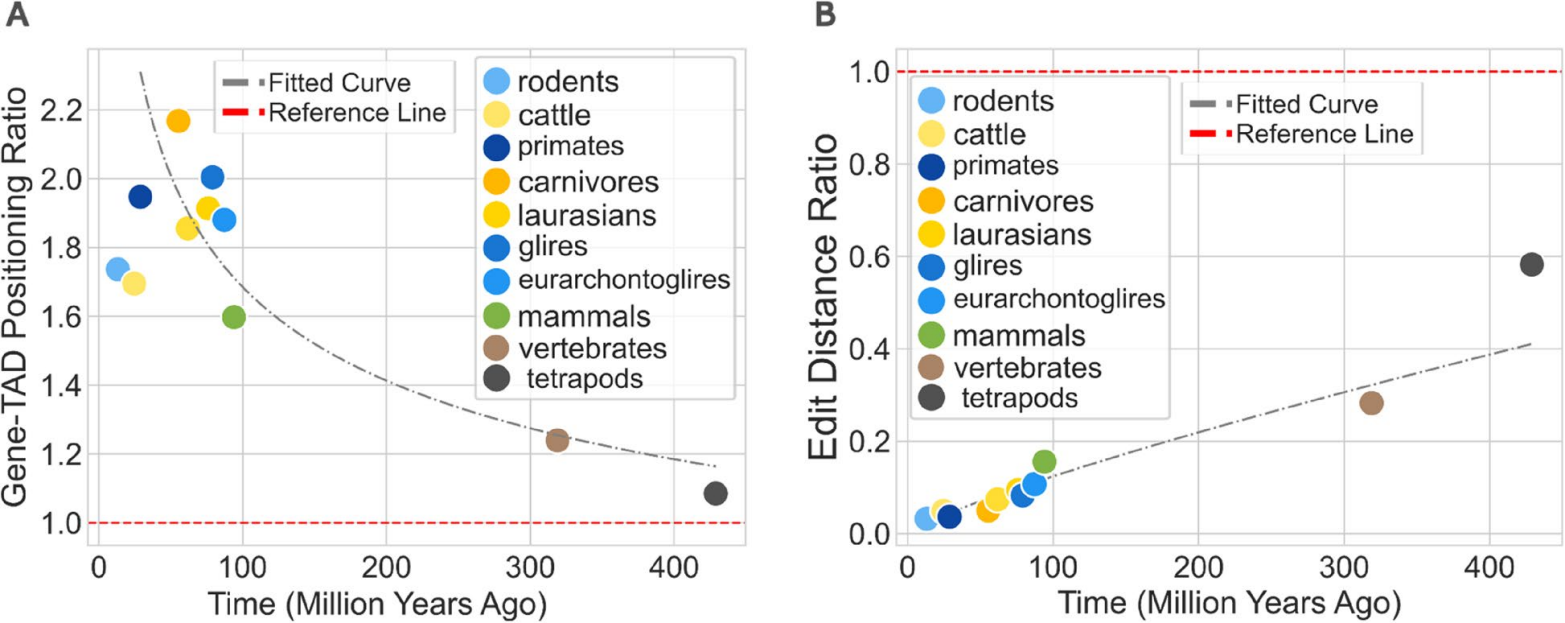
Syntenic gene analysis indicates stable gene-TAD conformation across vertebrate evolution. **A** Scatter plot showing the frequency of syntenic genes maintaining the same spatial localisation within TADs for blocks with matching TAD counts across different species pairs. The plot demonstrates that as evolutionary distance increases, the frequencies decrease, indicating greater conservation of gene spatial organisation in closely related species. **B** Scatter plot showing the relationship between the edit distance (TAD border insertions or deletions) and the evolutionary distance between species. The plot demonstrates that as evolutionary distance increases, more edits are needed, indicating a decrease in TAD conservation over time

To test if these levels of conservation were higher than expected by chance alone, we shuffled gene positions within each block 1000 times preserving the number of genes per block and the number of TADs, providing a baseline for comparison with our observed gene conservation scores. This revealed that genes generally maintained their spatial positioning across species, exceeding random expectation by factors ranging from 1.1 to 2.2 (Fig. 4A), with a *p* value < 0.05 (see Additional file 1: Fig. S10). For instance, in primates, gene positional conservation within TADs was observed to be 2 times higher than by chance (Fig. 4A), yet with a decreasing ratio over evolutionary time.

Next, we expanded these analyses to include syntenic blocks regardless of the number of TADs they contained. For this, we computed the theoretical number of TAD border insertions or deletions needed to ensure that orthologous genes resided within the same TADs between a pair of species.

The results show that the average number of required edits (i.e. insertions or deletions) is 0.8 borders/Mbp for mammals, increasing to 1.02 borders/Mbp in tetrapods and 1.08 borders/Mbp in vertebrates (Additional file 1: Fig. S11). The increasing number of required edits to convert one conformation into another correlates with the increasing evolutionary divergence between species. Computing the required border edits relative to shuffled genes (see above) revealed that all compared clades show a significantly lower number of border edits than expected by chance (Fig. 4B, Additional file 1: Fig. S12).

### Most syntenic blocks show stabilising selection while a few evolve under genetic drift

All analyses so far revealed a significant conservation of TAD features, yet with a strong phylogenetic signal. Hence, to assess whether TAD conservation is driven by stabilising selection or evolving under genetic drift, requires us to apply comparative phylogenetic models which can distinguish between these evolutionary forces. Previous studies of conservation of gene expression [34] have made use of the Ornstein–Uhlenbeck (OU) process, which is a widely accepted model of continuous trait evolution [34–36].

To analyse the evolutionary dynamics of TAD numbers within syntenic blocks, we used the OU model, which quantifies the influence of both random genetic drift and stabilising selection. It includes two parameters: alpha (*α*), indicating the strength of selection pressure, and sigma (*σ*), representing the rate of genetic drift. A larger *α* value suggests strong stabilising selection that pulls a trait towards an optimal value, while a larger *σ* value points to greater variability due to random changes. By applying the OU model, we assessed how TAD number evolution is influenced by these factors in mammals. We focused on mammals in our analysis due to the availability of Hi-C data for these species, which provided a more robust dataset for our study. The representation of other vertebrate species in the dataset is limited, with only two species from outside the mammalian group. Including these outgroup species would have further reduced the number of genomic regions available for analysis, limiting the statistical power of our results.

**Fig. 5:**
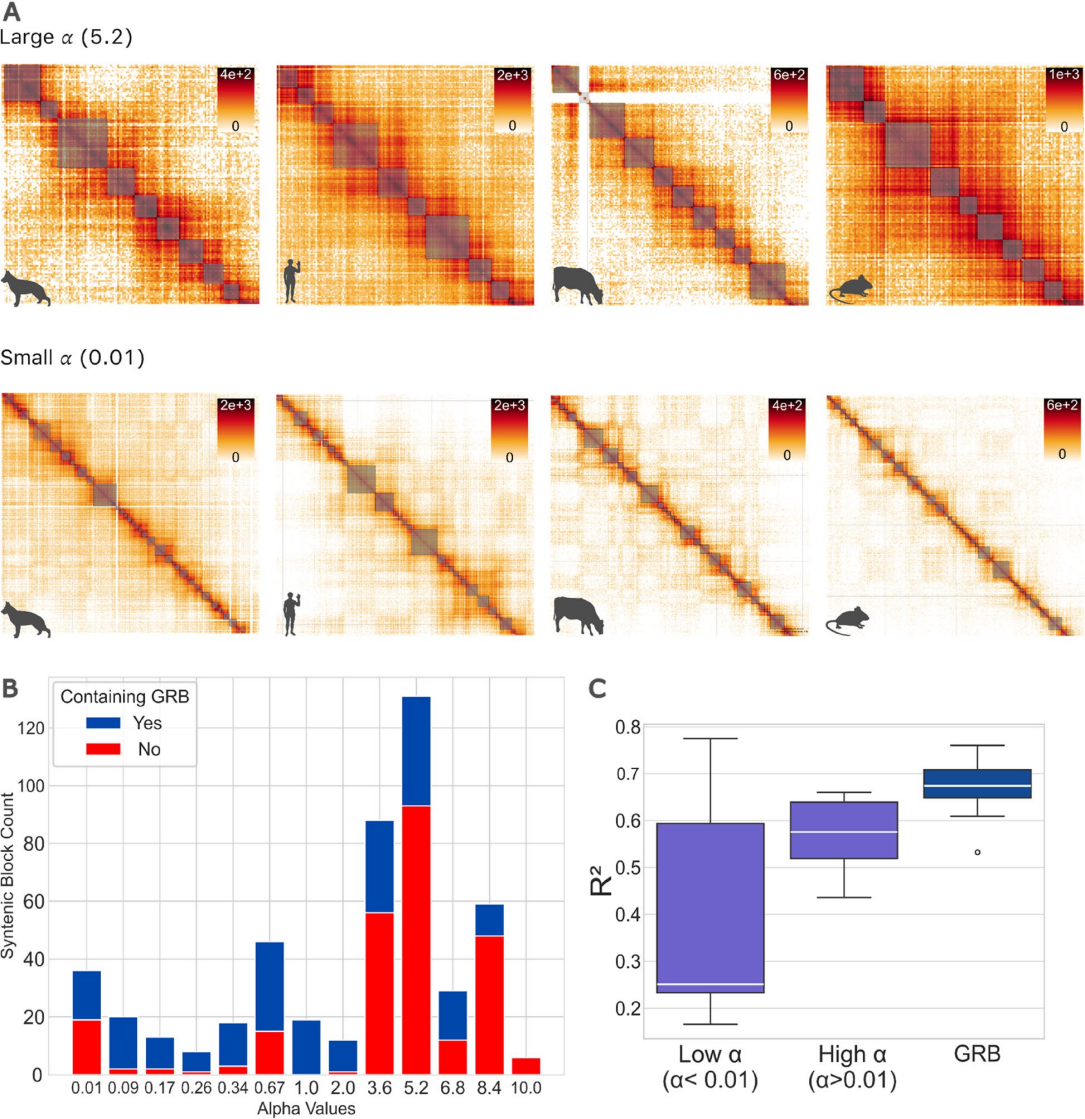
Ornstein–Uhlenbeck analysis uncovers stabilising selection on TAD numbers in vertebrates. **A** Heatmap showing 4 species (dog, human, cow, and mouse) for both large and small *α* values. **B** Stacked histogram of *α* values, where the blue section represents the fraction of syntenic blocks with at least one GRB and the red section represents blocks without any GRB. Most blocks show large *α* values, suggesting rapid convergence to an optimal TAD number and strong stabilising selection. A smaller subset of blocks with lower *α* values indicates weaker selection, approaching the Brownian motion model. **C** Boxplot displaying the distribution of *R*^2^ values for blocks with low and high *α* values, as well as for blocks containing at least one GRB, regardless of *α* value

We performed a grid search across a range of *α* and *σ* values, selecting the best-fitting parameters based on log-likelihood (see Methods). As *α* approaches zero, the process mimics Brownian motion, pointing to genetic drift, while higher *α* values suggest the presence of selective pressures. The cumulative log-likelihood results show a range of optimal *α* and *σ* parameters, primarily concentrated in regions with large *α* values (Additional file 1: Fig. S13), indicating significant selective pressure on many syntenic blocks. A smaller peak at low *α* values indicates that some TADs may be evolving under near-neutral conditions. These patterns highlight the diverse evolutionary dynamics across different syntenic blocks (Fig. 5A). Our analysis identified the most frequent parameter combination as *α* = 5.2 and *σ* = 2.11 (Additional file 1: Fig. S14), suggesting strong selection and low variability due to genetic drift, suggesting that stabilising selection plays an important role in conserving TAD numbers over evolutionary time. Interestingly, 4.12% syntenic blocks displayed *α* < 0.01, indicating that their evolutionary trajectories are more affected by genetic drift. Performing GO analysis on blocks with low *α* (*α* = 0.01) reveals terms like collagen catabolic processes (adjusted *p* = 0.02; Additional file 1: Fig. S15A), extracellular matrix disassembly (adjusted *p* = 0.02), and regulation of neuroinflammatory response (adjusted *p* = 0.02). These enrichments are primarily explained by several genes in the *MMP* cluster on human chromosome 11 and *CD200* on human chromosome 3.

The majority of blocks (95.88% of blocks) are concentrated at higher *α* values (*α* > 0.01), indicating strong selection on TAD numbers (Fig. 5B). High-*α* blocks are associated with various processes like intermediate filament organisation, organic acid transport, and glycerophospholipid metabolic process (Additional file 1: Fig. S15B). Noteworthy terms are also those related to development, including pattern specification, limb development, and gliogenesis. Many syntenic blocks under stabilising selection (high *α* values, ~ 8000 genes) contain developmental genes, while fewer blocks evolving through genetic drift have diverse, non-developmental functions.

The presence of developmental genes in our high-*α* blocks suggests that regulatory regions surrounding these genes could be relevant for the conservation of TAD structure. Highly conserved noncoding elements (CNEs) are known to surround developmental genes in vertebrates [37]. Clusters of these CNEs, termed genomic regulatory blocks (GRBs) [27], have been shown to overlap with TAD structure, suggesting that TADs are needed to maintain the regulatory architecture in these regions [26]. To investigate whether our synteny blocks associate with GRBs, we overlapped them with GRBs from reference [26]. While GRB-containing syntenic blocks span a range of *α* values, they do not strongly correlate with *α* (Fig. 5B). Our low-*α* blocks generally show a less conserved TAD border positioning compared to high-*α* blocks, confirming that values of *α* reflect levels of TAD border position conservation (Fig. 5C). Notably, GRB-containing blocks tend to have even more stable TAD border positions compared to high-*α* blocks in general (Fig. 5C). GO term analysis reveals that GRB-containing low-*α* blocks are not enriched for a particular process (Additional file 1: Fig. S15C; adjusted *p* > 0.35), and high-*α* blocks are mainly enriched with various developmental processes (adjusted *p* values < 2.52e − 04; Additional file 1: Fig. S15D). This confirms that the subset of GRBs with conserved TAD structures indeed harbour genes with developmental functions. Genes in non-GRB low-*α* blocks, on the other hand, are enriched in processes related to collagen metabolism (adjusted *p* values < 2.43e − 05; Additional file 1: Fig. S15E), whereas non-GRB high-*α* blocks are not enriched for particular processes (Additional file 1: Fig. S15F). This shows that syntenic blocks with conserved TAD numbers (high *α*) also harbour genes with non-developmental functions, especially if they do not harbour GRBs.

Comparing values of *α* with average number of genes and TADs within our blocks reveals that low-*α* blocks have a higher gene and TAD density than intermediate *α* values (0.09–6.8). For blocks with *α* > 8.4, gene and TAD density is the highest (Additional file 2: Table S5). Consistent with prior studies [26], TADs overlapping GRBs have lower gene density (e.g. 12.60 genes/Mbp in humans vs. 20.82 genes/Mbp in non-GRB TADs, Additional file 2: Table S6). However, protein sequence similarity between species remains comparable across low- and high-*α* blocks (Additional file 1: Fig. S16).

Overall, our analyses reveal that most syntenic blocks are governed by stabilising selection of their TADs. A subset of these which harbour GRBs are associated with developmental genes, consistent with prior studies. However, we also identify a large proportion of non-GRB blocks with conserved TAD structure harbouring genes with non-developmental functions.

## Discussion

TADs are essential functional units within the genome that play a significant role in regulating gene expression. Disruptions of TAD borders have been linked to abnormal gene interactions, gene mis-regulation, and various aberrant phenotypes, including cancer [38], and developmental disorders such as human limb malformations [39]. TADs play a crucial role in shaping the regulatory landscapes of developmental genes [28]. TAD border conservation across metazoans suggests strong selective pressure to maintain gene regulation by restricting the reach of gene regulatory interactions [26, 40, 41].

Our study expands the current understanding of evolutionary conservation of TADs across a range of vertebrate species. We have extended the analysis of evolutionary conservation of TADs across 12 vertebrate species, using a comprehensive quantitative framework to assess TAD-feature conservation, including the use of phylogenetic models to detect signatures of selection on TADs. Our approach integrates both a general assessment of TAD conservation across species and a focused analysis of individual blocks using the OU model. The initial broad analysis provides essential context for understanding overall conservation patterns, while the OU model allows us to examine the evolutionary conservation of TAD numbers in greater detail. Early studies on TAD conservation primarily relied on indirect methods or limited locus analyses, such as the *Hox* [42] and *Six* [43] clusters. These investigations suggested TAD conservation based on patterns like the enrichment of rearrangement breakpoints at TAD borders and their depletion within TAD bodies across species [17, 18].

Previous studies in vertebrates have reported various levels of TAD conservation, ranging from 54 to 14% across mammalian lineages of different evolutionary divergence levels (2, 19, 20). Our findings indicate that conservation of TAD number, border positions, and gene positioning within TADs display a strong phylogenetic signal (Figs. 2A, 3A, 4). Comparing our findings directly with previous studies is challenging largely due to the lack of standardised methodologies for defining and computing TAD conservation. For instance, while some studies inferred conservation through CTCF-binding sites [16], without a formal testing against a robust null model, others focused on broader comparisons not limited to syntenic regions. These methodological differences, combined with variability in data quality, and in TAD calling algorithms [44], limit direct comparisons of conservation values between different studies. Nevertheless, our findings point towards conservation of TAD features during vertebrate evolution.

Despite the evidence for evolutionary conservation, previous studies have not addressed the question whether this conservation results from selection or genetic drift. In this study, we apply an OU model with parameters that can be interpreted as the force of stabilising selection (*α*) and genetic drift (*σ*), enabling this distinction (28, 29). Our results revealed that a majority (95.88%) of syntenic blocks among mammals exhibit *α* > 0.01 (Fig. 5B), indicating that TAD structure in genomic regions with conserved synteny evolves under stabilising selection. Genes in these blocks under stabilising selection were associated with developmental processes, including embryonic organ development and regionalisation, while blocks lacking signatures of stabilising selection were not enriched for developmental genes. This result reinforces the idea that TAD structures are critical for maintaining normal gene regulation during embryo development and less so for non-developmental genes. Achieving accurate parameter estimates in OU models can be challenging, especially when tree size is small (< 100) [45]. However, our study does not hinge on individual parameter estimates. Instead, we analyse the distribution of *α* values across hundreds of synteny blocks, revealing that most synteny blocks exhibit *α* values much greater than sigma. This strongly suggests the presence of stabilising selection acting on the TAD number.

TADs associated with GRBs tend to exhibit lower gene density and higher border conservation, as developmental TADs are typically gene-poor but rich in intergenic regions that provide regulatory flexibility [26]. In contrast, TADs located further from GRBs often have higher gene density and greater structural variability, likely supporting specialised, less conserved processes [26]. However, GRBs are not the sole factor driving TAD conservation. Some GRB-free TADs remain highly conserved, displaying higher-than-average border similarity and strong stabilising selection on their number. This suggests that while GRBs contribute to TAD conservation, especially for developmental genes, their presence is not always necessary for maintaining TAD structural integrity. Selective pressure to preserve 3D genome organisation and regulatory interactions is possible even when enhancer sequences diverge, as many enhancers function through flexible, degenerate TF binding motifs—allowing regulatory activity to be maintained despite limited sequence conservation [46, 47]. TAD conservation could also be driven by other molecular processes. For instance, the TAD structures may serve as a 3D scaffold that optimises long-range interaction, without being essential to establish them. Some experimental evidence shows that depletion of CTCF or cohesin leads to a dramatic loss of TADs, yet with only modest changes in gene expression [48, 49]. Beyond gene regulation, other processes could also play a role in preserving TAD structures, such as timing of DNA replication or DNA repair [50].

Gathering large-scale Hi-C data from the same tissue or cells across multiple species complicates large-scale comparative studies. Additionally, teleost genomes like zebrafish, which experienced a third whole-genome duplication (3WGD), add a level of complexity that our analysis does not fully address. While genomes from non-3WDG species such as sharks and holosteans offer a potential alternative, the available Hi-C datasets for these species originate from different tissues and lack the resolution required for meaningful comparison. Analysing data from more species using similar approaches as applied here will further enhance our understanding of shared patterns and lineage-specific differences in 3D genome conservation. Moreover, comprehensive mapping of conserved 3D genome structures can pinpoint regions and TADs vulnerable to disease upon disruption.

## Conclusions

Our study enhances the current understanding of TAD conservation across a range of vertebrate species by employing a nuanced approach that includes various TAD features. We found that TAD numbers, border positions, and gene locations exhibit substantial conservation. Using the Ornstein–Uhlenbeck model, we identified signatures of stabilising selection on TAD numbers in many syntenic blocks. A subset of these blocks overlaps with GRBs and harbours developmental genes, highlighting the importance of maintaining precise TAD configurations for crucial gene regulatory functions. Conversely, genes involved in specialised functions showed signs of TAD evolution primarily driven by genetic drift, suggesting TAD organisation is less critical for these genes. Our findings underscore the evolutionary stability of TADs, while also illustrating the need for standardised methodologies in assessing TAD conservation.

## Methods

### Data sets and synteny identification

In this study, we analysed 12 different species: human (GRCh38.p14), rhesus macaque (Mmul_10), mouse (GRCm39), rat (mRatBN7.2), rabbit (UM_NZW_1.0), cow (ARS-UCD1.3), sheep (ARS-UI_Ramb_v2.0), pig (Sscrofa11.1), cat (F.catus_Fca126_mat1.0), dog (Dog10K_Boxer_Tasha), chicken (bGalGal1.mat.broiler.GRCg7b), and zebrafish (GRCz11). To investigate the relationship between synteny and 3D chromatin architecture, we first identified syntenic blocks using the Cyntenator tool. The three primary input files for this tool are:

- Guide tree: specifies the order in which the genomes should be aligned.
- Genome file: contains coordinates for protein-coding genes.
- Correspondence file: details orthologous relationships among genes across all input genomes.

The correspondence file was generated by performing an all-versus-all alignment of protein sequences from the 12 species using mmseq2 [51] with an e value threshold of 10−5. Only the longest isoform from each protein sequence was kept. The phylogenetic tree from the TimeTree database served as the guide tree for progressive alignment. Cyntenator uses a stepwise alignment strategy, gradually adding species based on their evolutionary relationships. This method results in alignments that capture syntenic blocks shared among species within a specific node of the guide tree. Essentially, the syntenic blocks identified at each internal node represent genomic regions conserved among species branching from a common ancestor.

### Processing and analysis of Hi-C data

Raw Hi-C sequencing data was obtained from the Gene Expression Omnibus (GEO: GSE167581, SRA IDs: PRJNA482496, [30, 31]) and processed using the nf-core/hic (v2.0.0) pipeline [52] to ensure quality control, sequence mapping, and filtering. The reference genomes utilised were the latest versions available as of 2023.

The pipeline was executed with HindIII as the restriction enzyme and was configured with the following optional flags: –skip_maps, –skip_dist_decay, –skip_tads, –skip_compartments, –skip_balancing, –skip_mcool, and –split_fastq = false.

For creating Hi-C contact matrices, we employed the Cooler software [53] (v0.8.11) to produce matrices in the cooler format. These matrices were subsequently converted to the. mcool file format using the cooler *zoomify* tool. Matrix balancing was then performed using iterative correction (ICE). Data from biological replicates were merged for comprehensive analysis. To identify TAD borders at a 32-kb resolution, we used the hicFindTADs command from HiCExplorer [32] (v3.7.2).

To identify TADs located within each syntenic block, we used BEDTools (v2.30.0) [54] *intersect*.

### Measuring TAD number conservation across vertebrates

To explore the relationship between synteny and the conservation of TADs throughout vertebrate evolution, we analysed various TAD characteristics across 12 different species. Our initial goal was to assess the conservation of TAD numbers within synteny blocks. First, we identified synteny blocks for each species and counted the TADs within them, normalising the counts by synteny block lengths in Mbp. We then calculated the absolute differences in these normalised TAD counts between species pairs, adjusting for evolutionary divergence times. By using linear regression to fit these differences against divergence times, we determined the slope, representing the average rate of TAD number change per Mbp per million years.

To further quantify the conservation of TAD numbers, we also used a scoring metrics defined:

- Average relative difference (ARD)

For each pair of species *i* and* j* within a given syntenic block *k*, the relative difference in TAD counts between species *i* and* j* within the block is:

$${RD}_{i,j}^{k}=\frac{{|T}_{i,k}-{T}_{j,k}|}{\frac{{T}_{i,k}-{T}_{j,k}}{2}}$$

The ARD score is the average of these relative differences across all syntenic blocks. This score quantifies the average relative difference in TAD counts between species *i* and* j* in the syntenic blocks. We created these randomised distributions by shuffling the order of syntenic blocks 1000 times. For each iteration, we calculated an ARD score and used the resulting distribution to evaluate the statistical significance of our observed ARD values.

### Quantifying the evolutionary conservation of TAD borders

We next focused on the positioning of TAD borders within synteny blocks to quantify their conservation throughout vertebrate evolution. We normalised the positions of TAD borders within synteny blocks on a scale from 0 to 1. This normalisation enabled us to compare TAD positions between different species using a consistent scale, regardless of their specific genomic coordinates. We assessed the changes in TAD borders positions over evolutionary time by calculating the absolute differences between corresponding borders within each synteny block, expressed as changes per million years. The species were grouped into three distinct evolutionary clades: mammals only, mammals and chicken, and a broader group including mammals, chicken, and zebrafish. For each group, we computed the average difference in TAD borders positions and analysed these differences in relation to species divergence. To further evaluate the conservation of TAD borders, we used the *R*^2^ score. This statistical measure quantifies the extent to which TAD borders in one species can predict corresponding borders in another species.

### Gene-TAD conformation stability evaluation

To evaluate the maintenance of syntenic genes within the same TAD across evolution, we focused on examining the positioning of orthologous syntenic genes within TADs to determine if they exhibit a tendency to reside within the same TAD. We computed the percentage of genes within the same TAD as the observed data, followed by a random permutation of TAD borders to establish a comparative baseline. This score was evaluated only for syntenic blocks with the same number of TADs. To further analyse the differences in TAD configurations between species, we introduced another score-defined edit distance. This score evaluates the number of edits—specifically, TAD border insertions or deletions—needed to transition from one TAD conformation to another.

### Ornstein–Uhlenbeck (OU) model

In evolutionary biology, modelling how traits evolve within populations over time is essential for understanding adaptation and divergence mechanisms. We employed the OU process to model the evolution of continuous traits along a phylogeny. Specifically, we analysed the number of TADs in each syntenic block across a mammalian alignment. Each syntenic block was analysed individually to assess how the number of TADs evolves over time in relation to the phylogeny.

The OU model is governed by the following stochastic differential equation (SDE):

$$dXt=-\alpha (Xt-\vartheta )+\sigma Bt$$

where *Xt* represents the trait value (in this case, the number of TADs) at time *t*, $\alpha$ is the rate of reversion to the mean trait value $\vartheta$, $\sigma$ denotes the strength of the trait’s stochastic variation, and *Bt* is a standard Brownian motion.

To estimate the parameters of this model, we compute the covariance matrix* V*:

$${V}_{i,j}=\frac{{\sigma }^{2}}{2\alpha }\left[{e}^{-\alpha {t}_{i,j}}(1-{e}^{-2\alpha {t}_{ra}})\right]$$

Here:

- *t*_*ij*_ is the time between the *i*th and *j*th tip of the mammal phylogeny.
- *t*_*ra*_ is the time from the root to the tip.

The design matrix *C* captures the expected trait values at the phylogenetic tips and is given by:

$${C}_{i1}={e}^{-\alpha {t}_{ia}}, {C}_{i2}={1-e}^{-\alpha {t}_{ia}}$$

Given the design and variance matrices, the parameters $\vartheta$ can be estimated by generalised least squares (GLS). GLS minimises the weighted sum of squared residuals, yielding the parameter vector:

$$\vartheta ={({C}^{T}{V}^{-1}C)}^{-1}{C}^{T}{V}^{-1}y$$

The trait values—TAD numbers in syntenic blocks—are extracted for each species and aligned according to the phylogenetic tree. For the parameter estimation, we performed a grid search over a range of values for $\alpha$ and *σ*. The grid search covered *α* and *σ* values from 0.01 to 10, allowing us to find the optimal set of parameters that maximised the log-likelihood function. This process helped us identify the best-fit model for trait evolution in syntenic blocks across the mammalian phylogeny.

### Gene Ontology enrichment analysis

We performed Gene Ontology (GO) biological process enrichment analysis using the clusterProfiler R package with the enrichGO function [55]. Terms with an adjusted *p* value < 0.05, calculated using the Benjamini-Hochberg (BH) method, were considered significantly enriched. The top 10 enrichment terms, ranked by their gene ratio in descending order, were selected for visualisation.

### GRB-including syntenic blocks identification

We obtained the list of GRBs from [26], which have identified using CNEs showing 70% identity over 50 bp between human and chicken (hg19-galGal4). Their genomic coordinates to the hg38 assembly using the UCSC LiftOver tool [56] with default parameters. To classify syntenic blocks as either GRB-including or non-GRB-including, we assessed their overlap with GRBs using bedtools intersect [54] with no filtering based on the extent of overlap.

### Identification of non-syntenic paralogy

To classify genes into possible non-syntenic paralogs and not, we analysed alignment blocks in the synteny dataset. For each block, we identified human genes absent in vertebrate alignment but present in tetrapods. These genes were checked against sequence similarity data to determine if alternative orthologs existed in zebrafish with no sequence identity, at least 70% sequence identity, and at least 80% sequence identity. Genes from the tetrapods exclusive set that had a significant match in zebrafish based on sequence similarity have been classified as non-syntenic paralogs or not.

## Supplementary Information

Additional File 1: Figures S1–S16. Fig. S1 GO enrichment in vertebrate syntenic regions. Fig. S2 Whole-genome Hi-C contact maps. Fig. S3 TAD number and distribution in the syntenic blocks. Fig. S4 TAD number difference by divergence time. Fig. S5 Random vs. observed ARD distribution per clade. Fig. S6 TAD border by divergence time. Fig. S7 Species-specific tad border differences and contact maps for SIX 2/3. Fig. S8 TAD borders correlation in primate syntenic blocks and enriched GO terms in lowly mapped borders. Fig. S9 TAD-gene conformation by divergence time. Fig. S10 Random vs. observed gene-TAD positioning distribution per clade. Fig. S11 TAD edits per Mbp by divergence time. Fig. S12 Random vs. observed TAD edits per Mbp distribution per clade. Fig. S13 Cumulative log-likelihood heatmap. Fig. S14 Heatmap of counts for alpha-sigma pairs. Fig. S15 GO terms by alpha value and GRB presence. Fig. S16 Average protein sequence identity.

Additional File 2: Tables S1–S6. Table S1 Summary statistics of syntenic blocks. Table S2 Summary of missing genes in vertebrate alignment for tetrapods. Table S3 Coverage of TADs within syntenic blocks across clades. Table S4 *R*^2^ and *p* values for TAD border comparison. Table S5 Syntenic blocks statistics across different *α* values. Table S6 Gene density in syntenic blocks with/without GRB.

## Data Availability

All data generated in this study and code used in this study have been submitted to Zenodo (https://zenodo.org/) and are available for download at the following DOI: 10.5281/zenodo.14628257 [57]. The available data include: 1).mcool files for the 12 species; 2) files containing the syntenic regions identified using Cyntenator across various clades; 3) TADs identified at 32 kb resolution for each species; 4) overlap between TADs and syntenic blocks across species.

## Abbreviations

3D: Three-dimensional
TAD: Topologically associating domain
OU: Ornstein-Uhlenbeck
CTCF: CCCTC-binding factor
GRB: Genomic regulatory block
CNE: Conserved noncoding elements
GO: Gene Ontology
Myr: Million years
ARD: Average relative difference
Mbp: Megabase pair
SD: Standard deviation
3WGD: Third whole-genome duplication
BH: Benjamini-Hochberg
GLS: Generalised least squares
SDE: Stochastic differential equation
