# Supplementary material for: TAD conservation in vertebrate genomes is driven by stabilising selection
Source: BMC Biol. 2025 Aug 5;23:241. doi: 10.1186/s12915-025-02362-0 (PMC12326840; doi:10.1186/s12915-025-02362-0)
Supplement: Supplementary file 1 — Additional File 1: Figures S1–S16. Fig. S1 GO enrichment in vertebrate syntenic regions. Fig. S2 Whole-genome Hi-C contact maps. Fig. S3 TAD number and distribution in the syntenic blocks. Fig. S4 TAD number difference by divergence time. Fig. S5 Random vs. observed ARD distribution per clade. Fig. S6 TAD border by divergence time. Fig. S7 Species-specific tad border differences and contact maps for SIX 2/3. Fig. S8 TAD borders correlation in primate syntenic blocks and enriched GO terms in lowly mapped borders. Fig. S9 TAD-gene conformation by divergence time. Fig. S10 Random vs. observed gene-TAD positioning distribution per clade. Fig. S11 TAD edits per Mbp by divergence time. Fig. S12 Random vs. observed TAD edits per Mbp distribution per clade. Fig. S13 Cumulative log-likelihood heatmap. Fig. S14 Heatmap of counts for alpha-sigma pairs. Fig. S15 GO terms by alpha value and GRB presence. Fig. S16 Average protein sequence identity. [file 12915_2025_2362_MOESM1_ESM.docx]

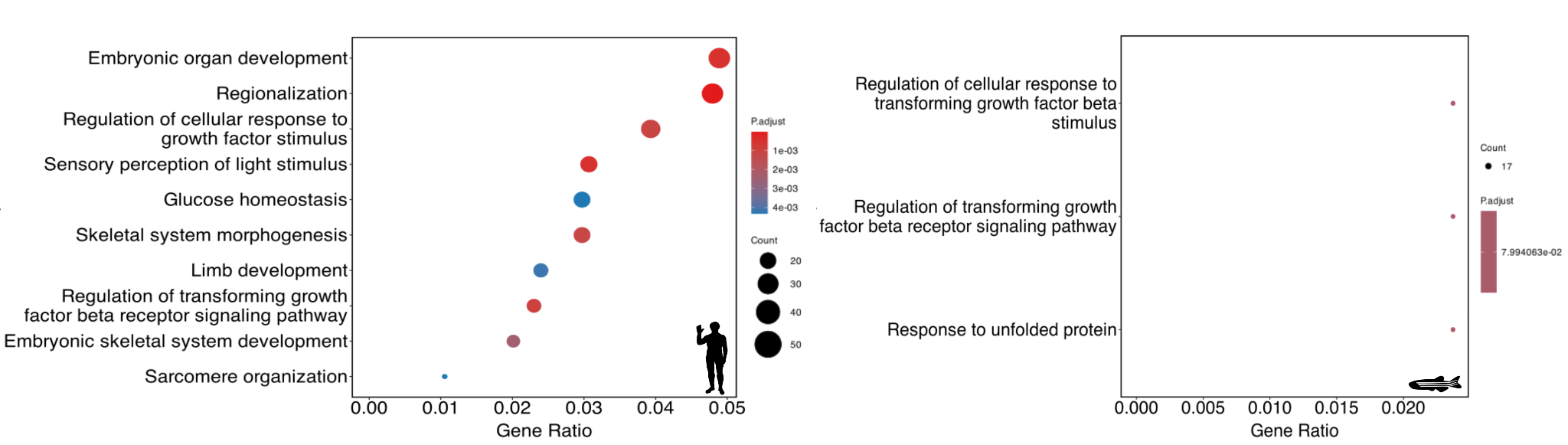


**Figure S1** GO terms enriched in vertebrates alignment.


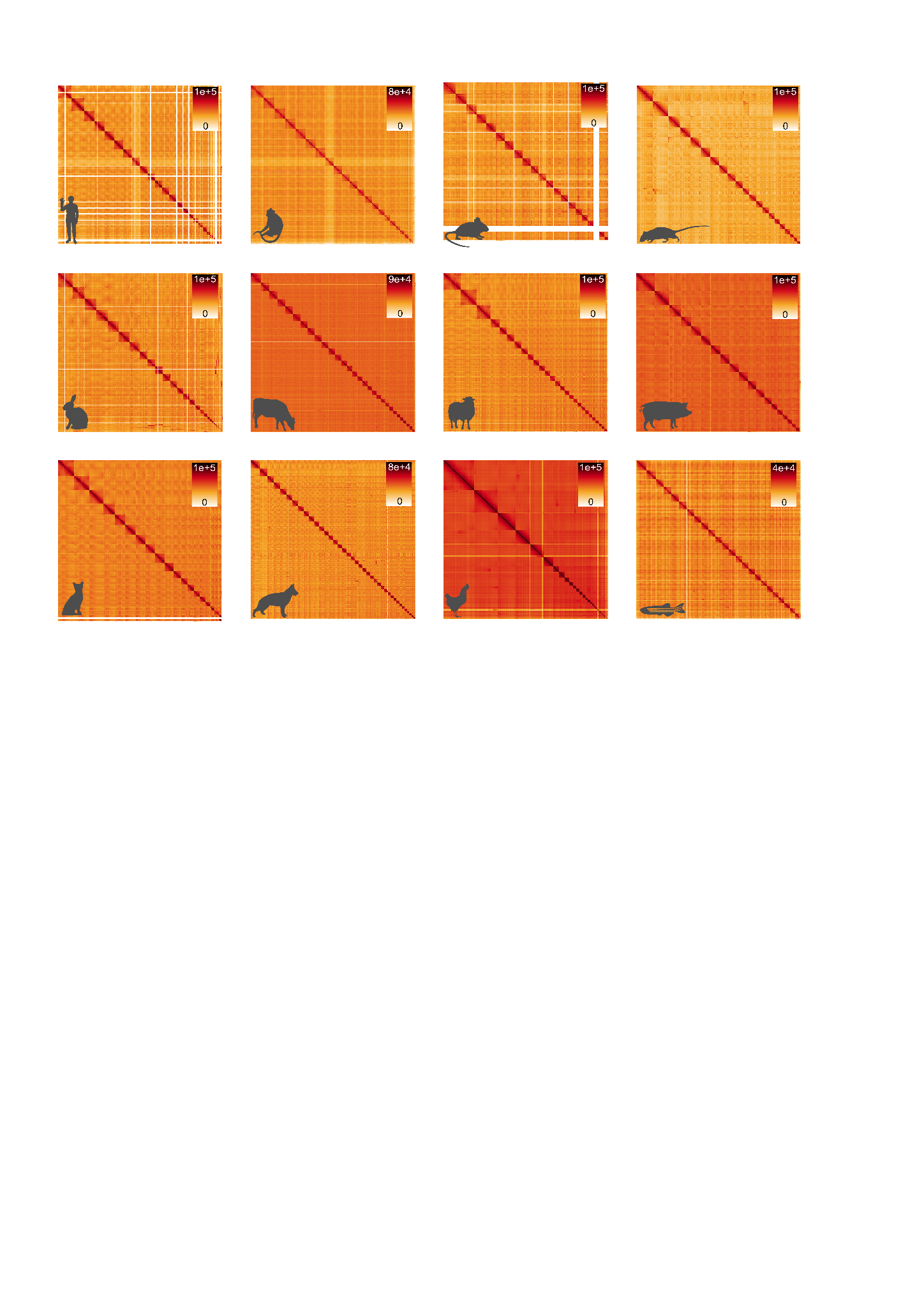


**Figure S2** Panel displaying Hi-C contact maps for the entire genome of each of the 12 vertebrate species included in the study.


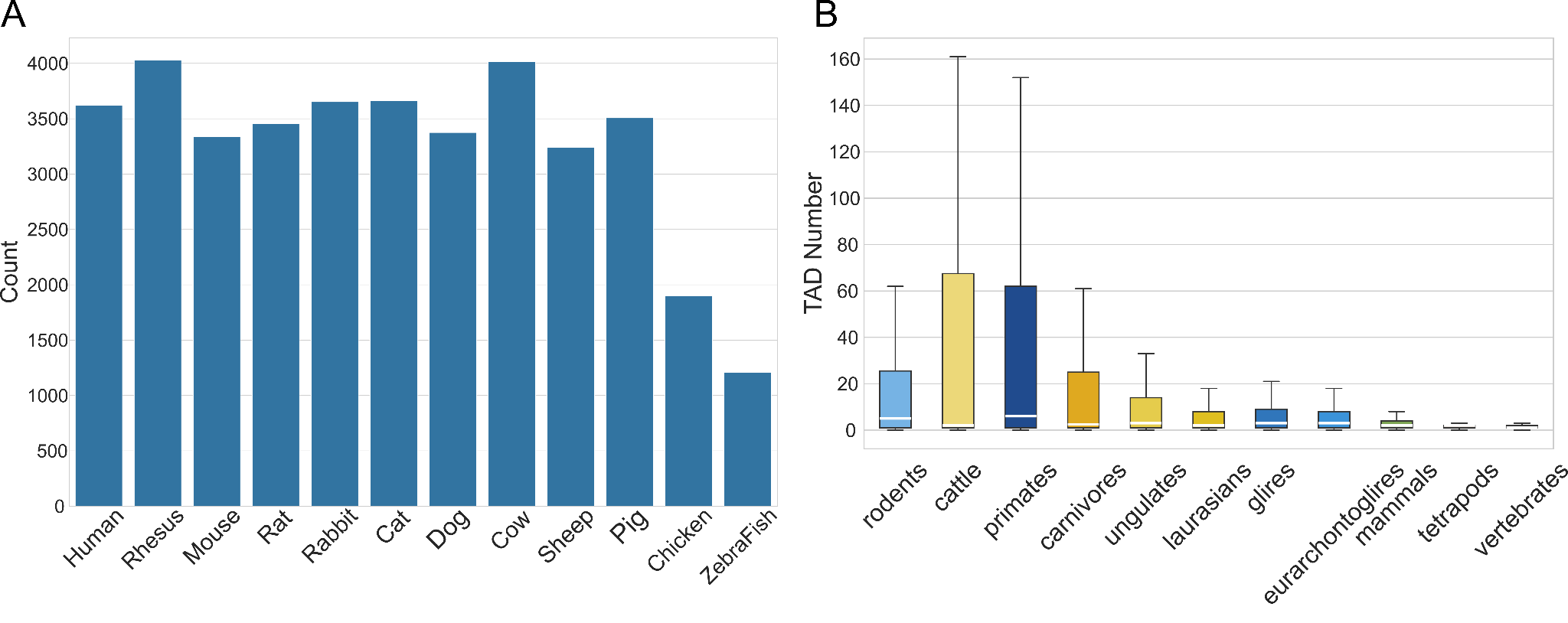


**Figure S3** **A** Total number of Topologically Associating Domains (TADs) identified in each species. **B** Distribution of TADs within syntenic blocks across different clades.


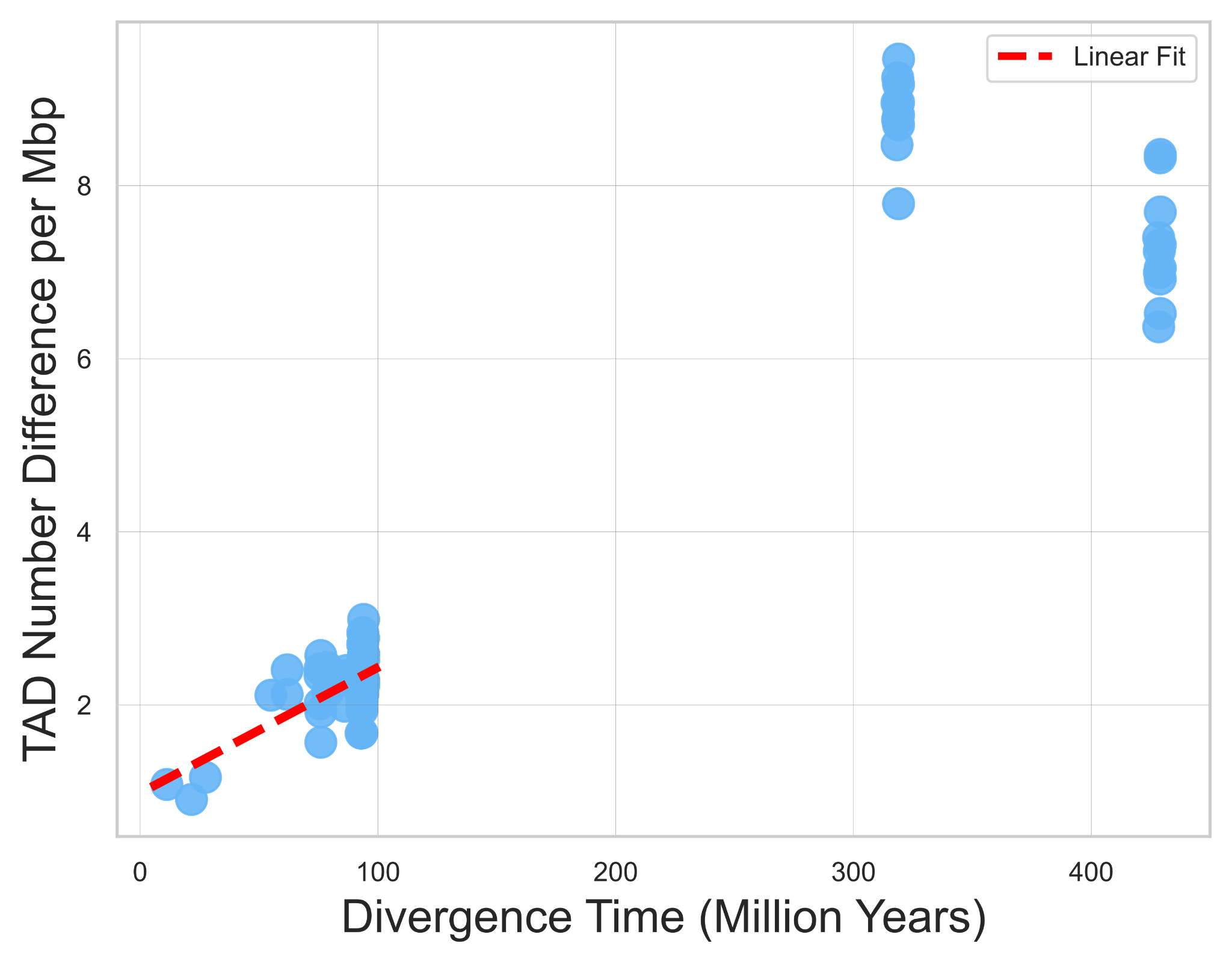


**Figure S4** Scatterplot showing the difference in TAD number per Mbp within syntenic blocks as a function of evolutionary distance between species. As evolutionary distance increases, differences in TAD number become more pronounced. The red dashed line represents linear fit to the mammalian species.

**
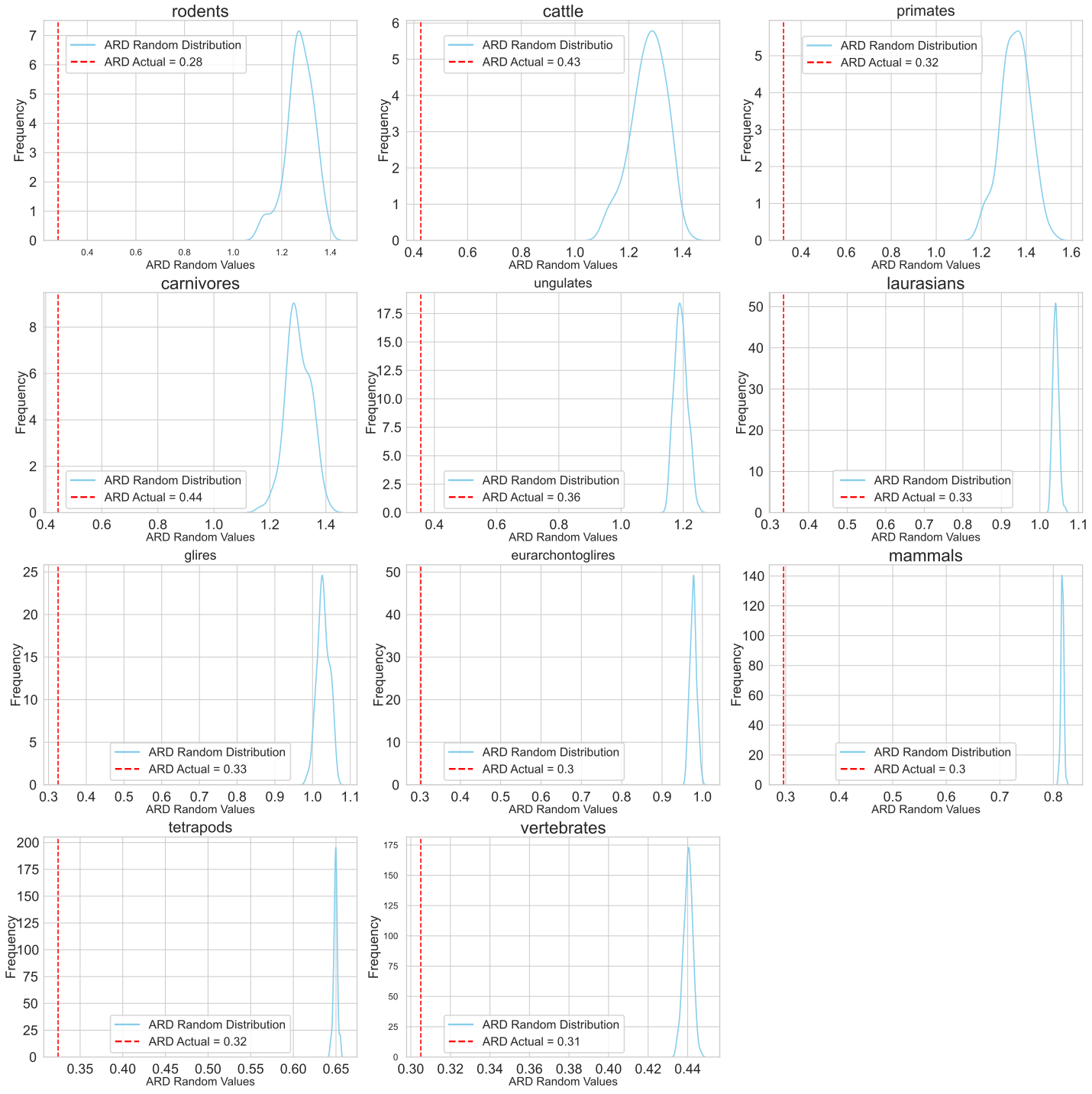
**

**Figure S5**  Distribution of 1,000 randomly generated ARD for each clade. The red vertical line represents the observed ARD value.


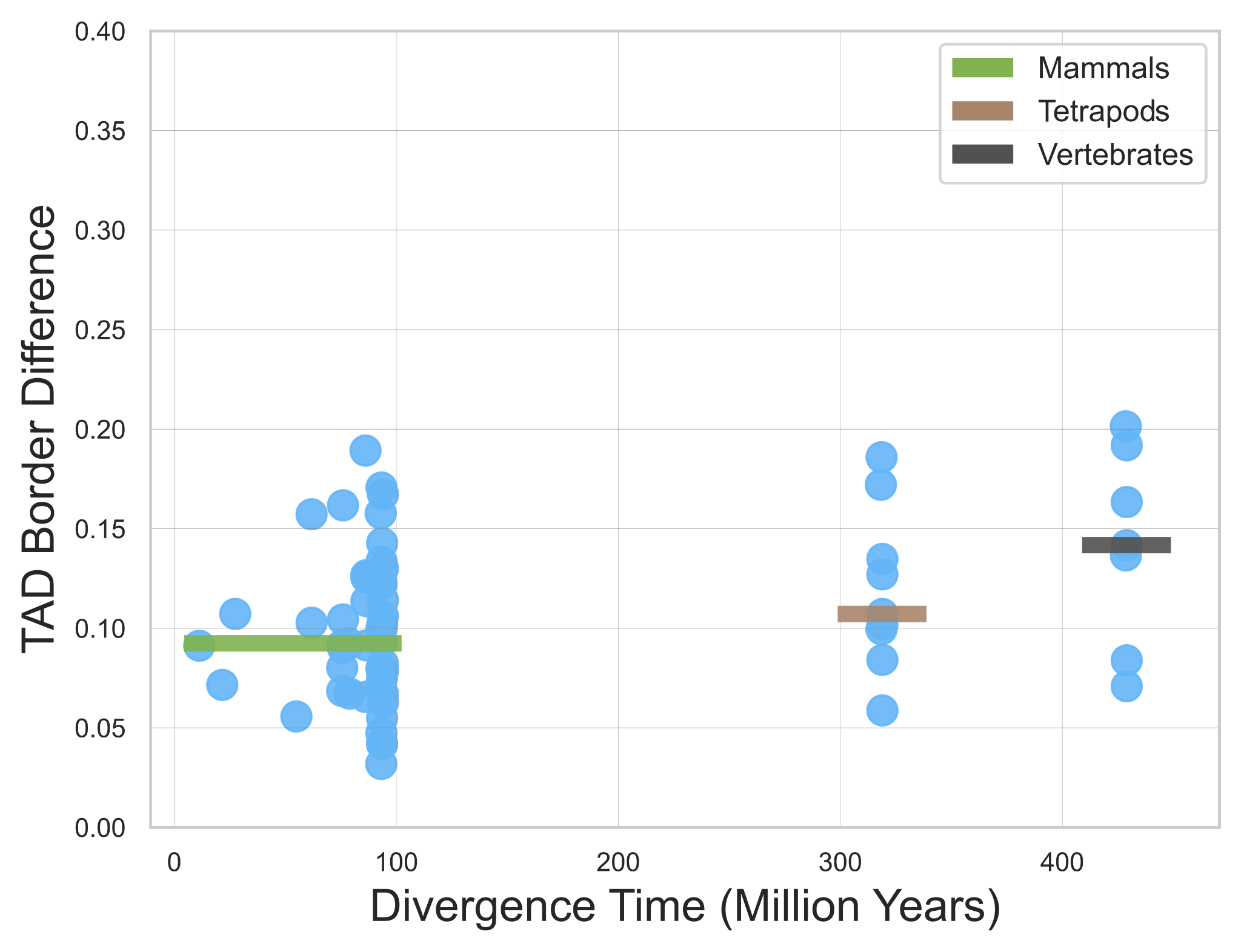


**Figure S6** Scatterplot showing the difference in TAD borders within syntenic blocks as a function of evolutionary distance between species. The three lines represent the mean difference in three different clades: mammals (green), tetrapods (brown), and vertebrates (black).

**
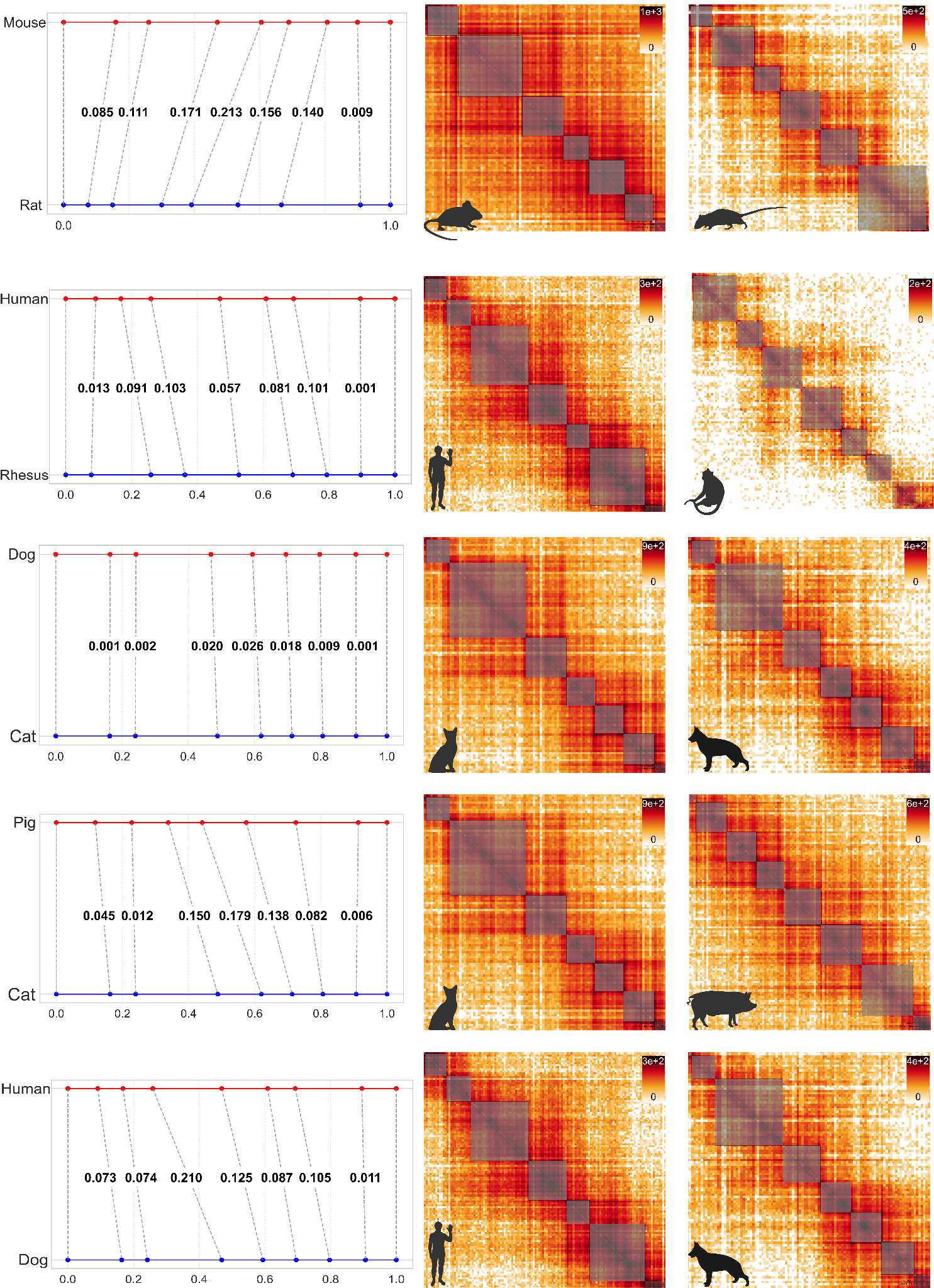
**

**Figure S7:** Comparison of the SIX2/3 cluster across different species pairs, including rodents, primates, carnivores, l aurasians, and mammals. Each row represents a distinct species pair from these clades. On the left side of the plot, TAD borders within the syntenic block are shown in the range between 0 and 1, along with the corresponding differences between the borders. On the right side, the contact matrices for each species in the pair.


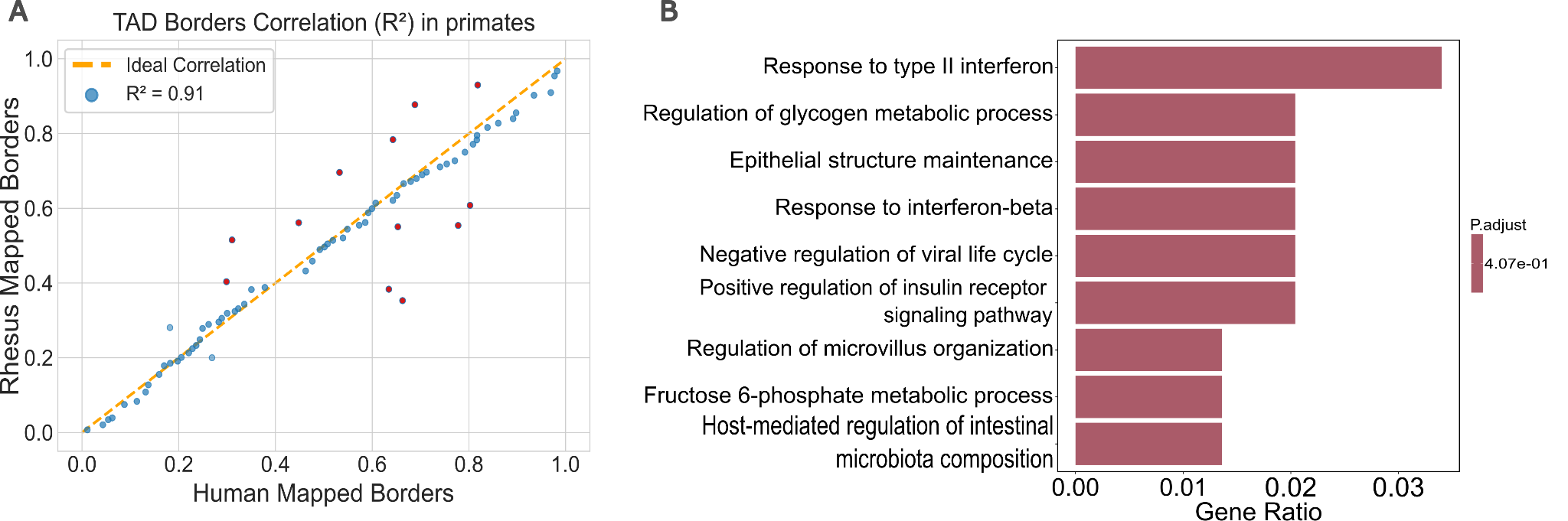


**Figure S8 A** Scatter plot showing all the TAD borders of syntenic blocks with the same number of TADs in the primates alignment. Red dots represent lowly mapped borders. **B** GO term analysis for genes in syntenic blocks associated with lowly mapped borders in primates. These GO terms are not significantly enriched.


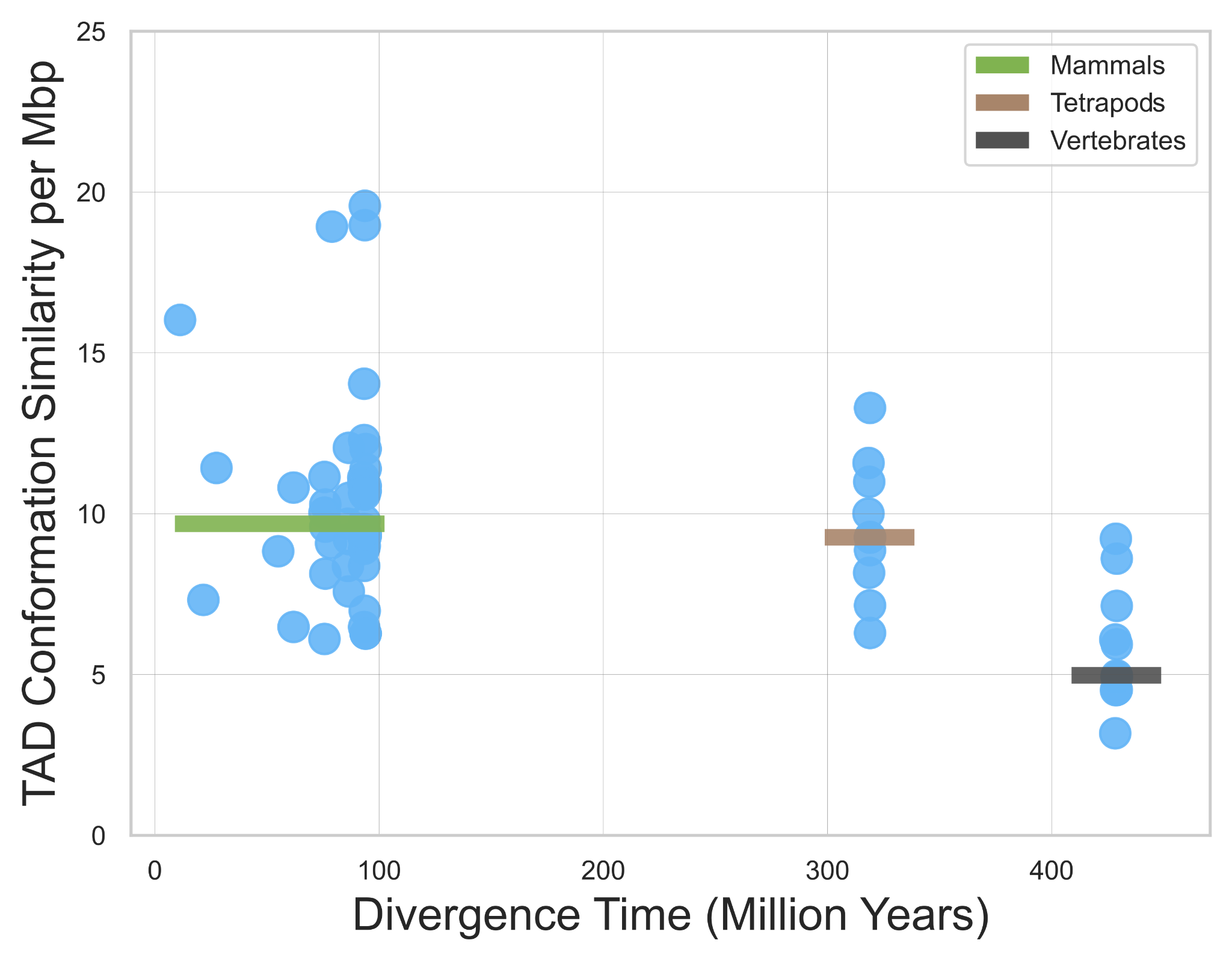


**Figure S9** Scatterplot showing the similarity in TAD-Gene conformation within syntenic blocks as evolutionary distance between species increases. The three lines represent the mean value in three different clades: mammals (green), tetrapods (brown), and vertebrates (black).


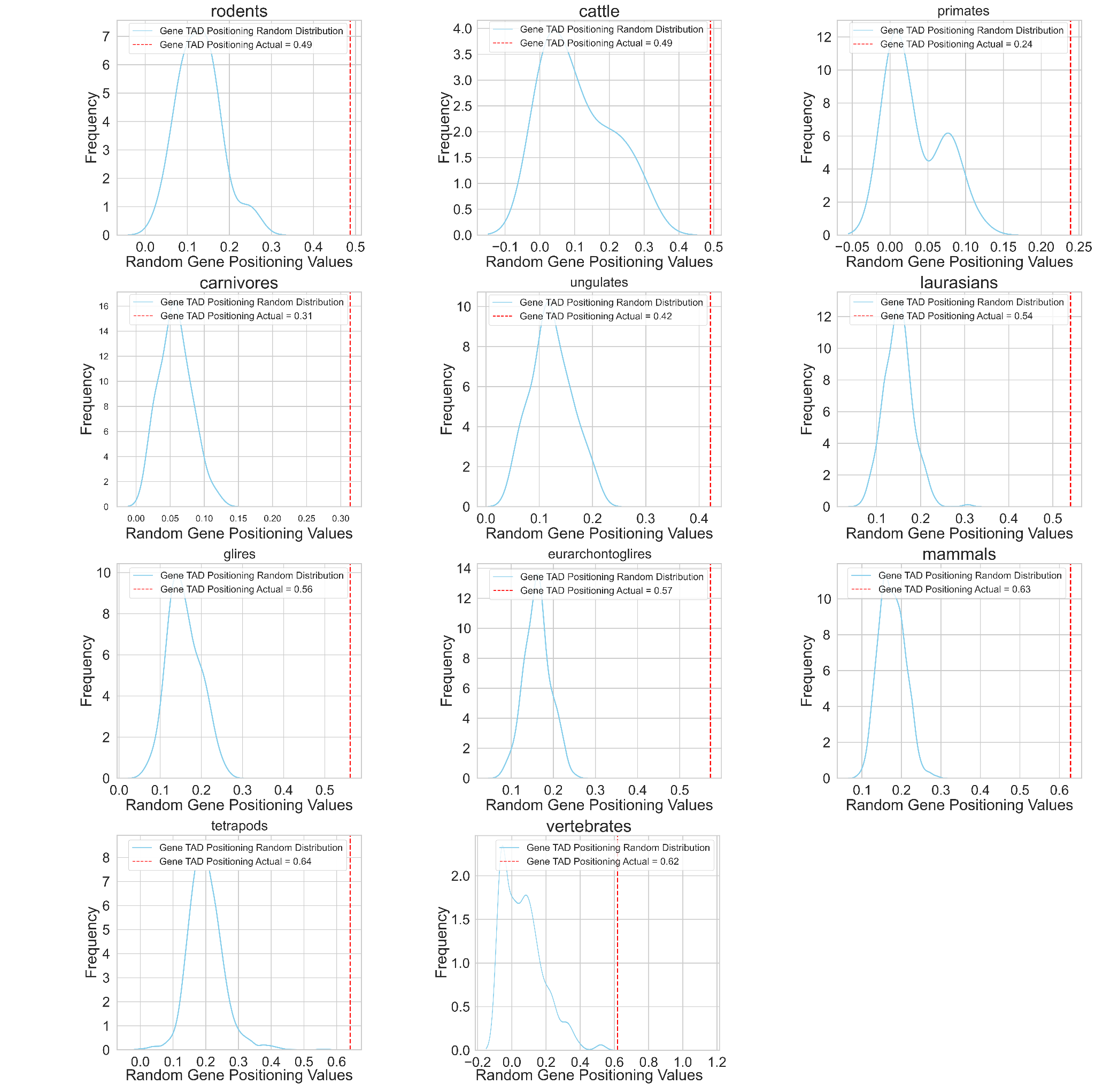


**Figure S10** Distribution of 1,000 randomly generated Gene-TAD Positioning for each clade. The red vertical line represents the observed Gene-TAD Positioning value.


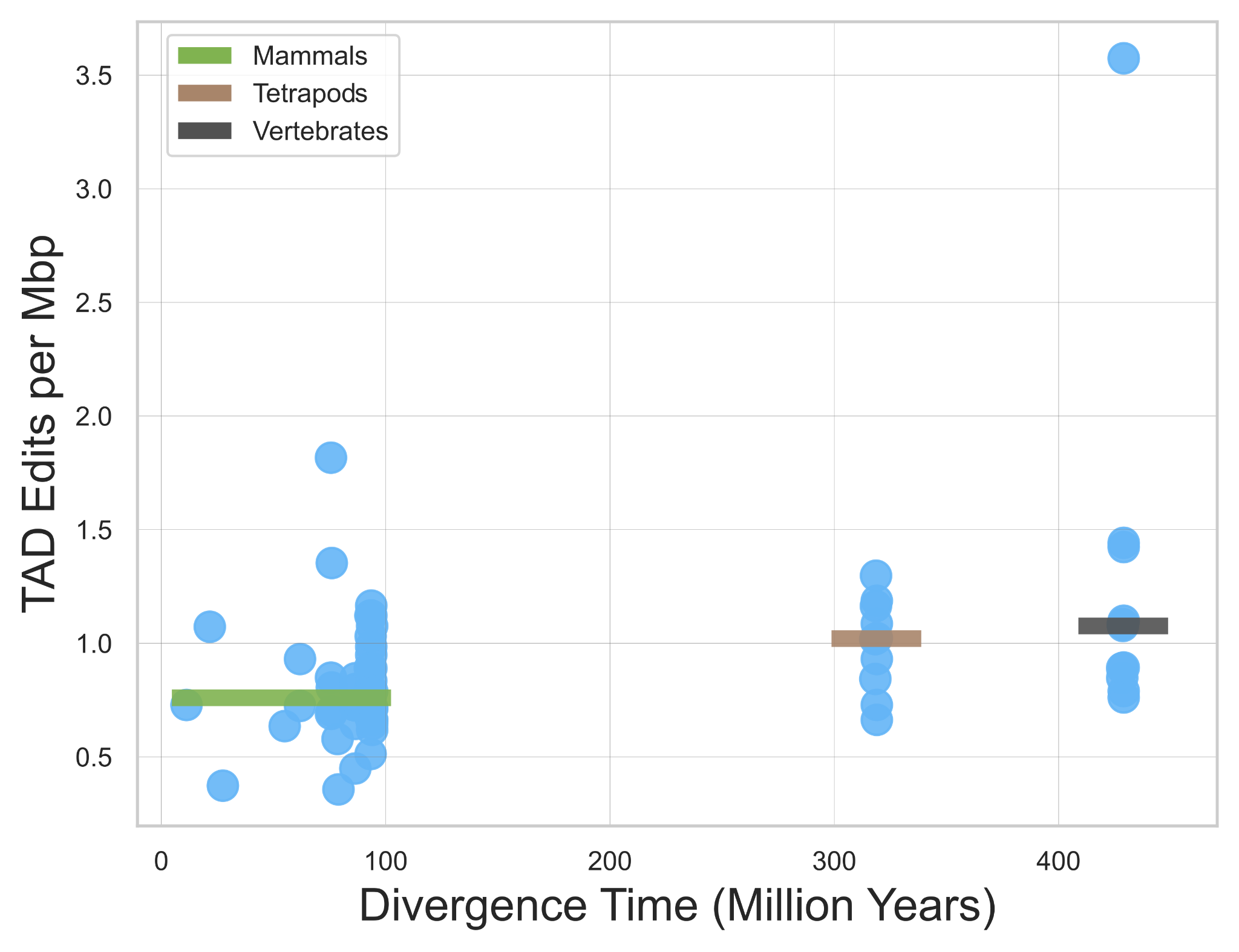


**Figure S11** Scatterplot showing the difference in TAD edits per Mbp within syntenic blocks as a function of evolutionary distance between species . The three lines represent the mean edits in three different clades: mammals (green), tetrapods (brown), and vertebrates (black).


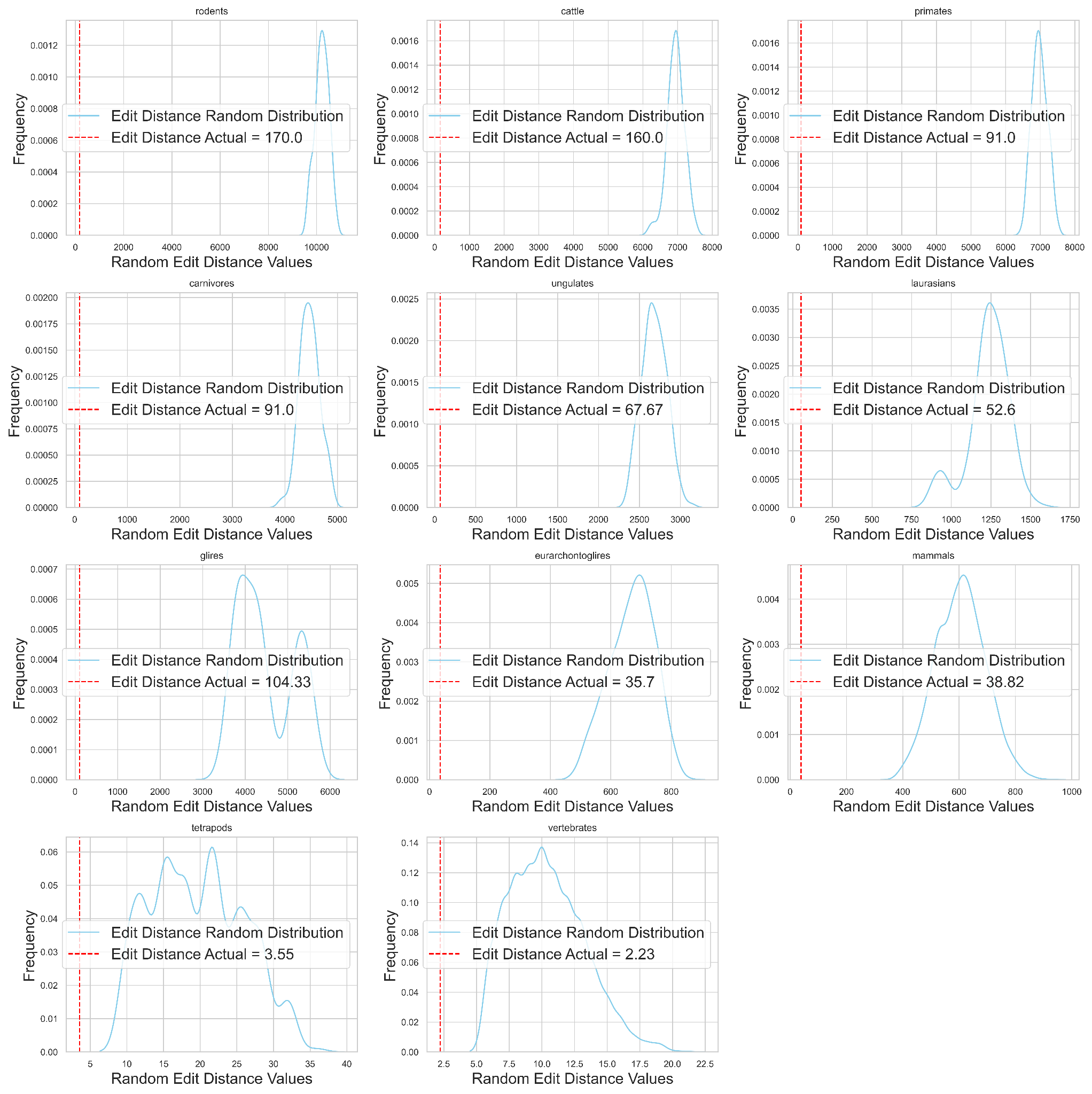


**Figure S12** Distribution of 1,000 randomly generated Edit Distance for each clade. The red vertical line represents the observed Edit Distance value.

**
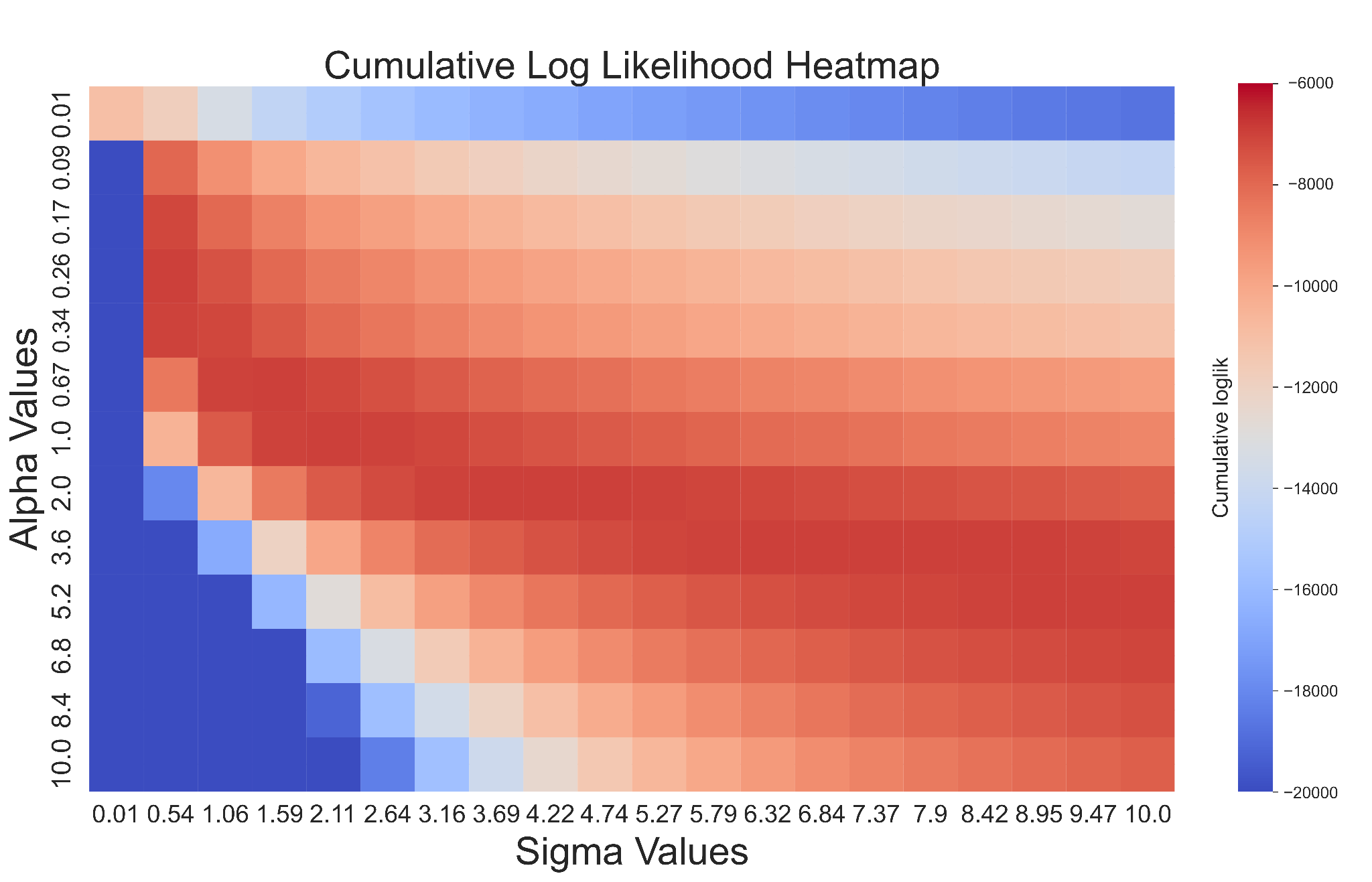
**

**Figure S13** Heatmap displaying the cumulative log-likelihood values for different alpha and sigma pairs.

**
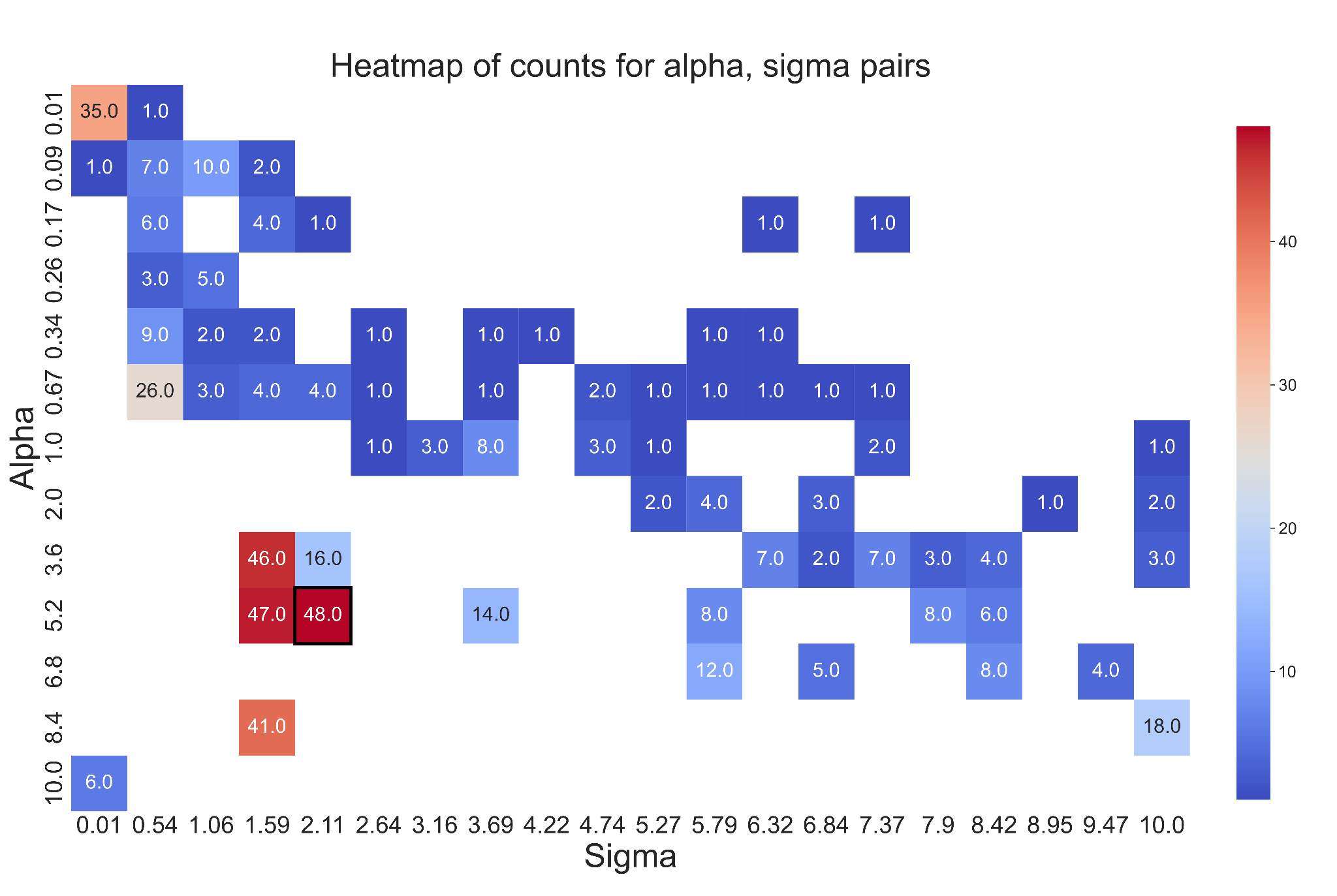
**

**Figure S14** Frequency of alpha and sigma pairs, with the optimal pair being (5.2, 2.11)

**
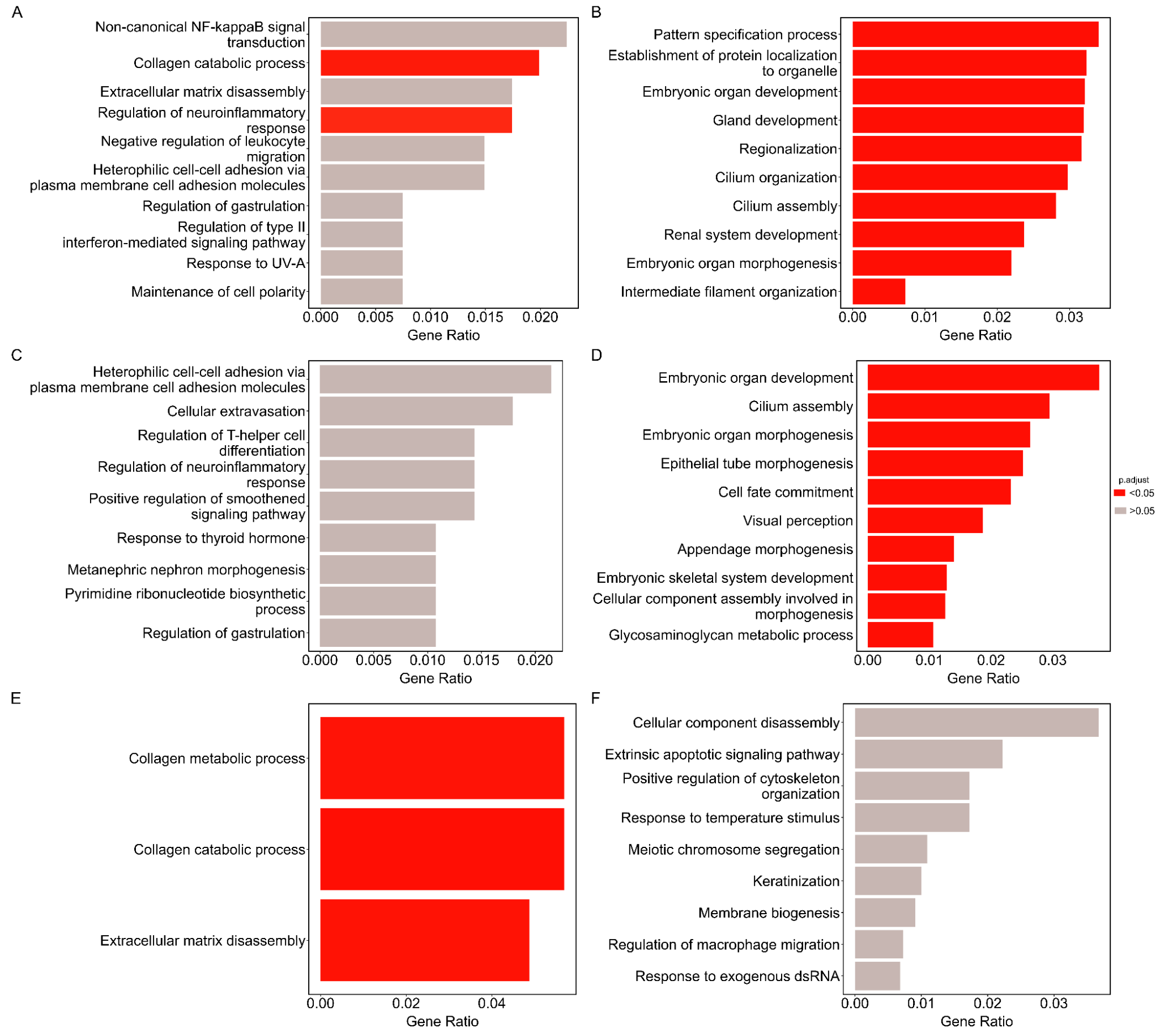
**

**Figure S15** GO term analysis for genes in syntenic blocks with different alpha values and the presence of GRBs. **A** GO terms for genes in low-alpha (0.01, 439 genes) syntenic blocks. **B** GO terms for genes in high-alpha (>0.01, 8092 genes) syntenic blocks.**C** GO terms for genes in low-alpha syntenic blocks with at least one GRB (306 genes). **D** GO terms for genes in high-alpha syntenic blocks with at least one GRB (5678 genes). **E** GO terms for genes in low-alpha syntenic blocks without GRBs (133 genes). **F** GO terms for genes in high-alpha syntenic blocks without GRBs (2415 genes). GO terms are ordered by gene ratio, and p-values are adjusted for multiple testing.


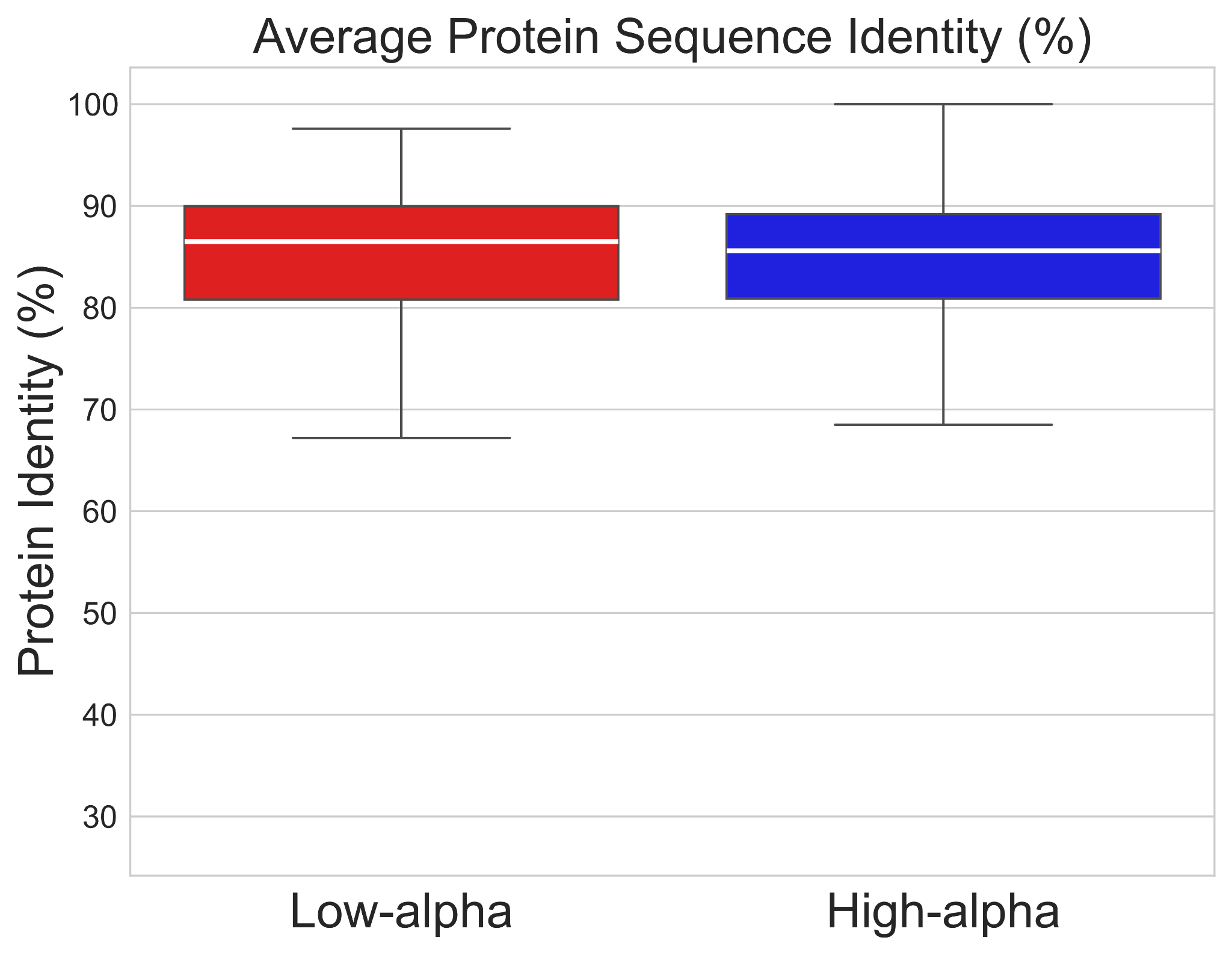


**Figure S16** The boxplot shows the distribution of average percentage identity of protein sequences between species for genes located in low-alpha (red) and high-alpha (blue) genomic blocks.
