# Supplementary material for: TAD conservation in vertebrate genomes is driven by stabilising selection
Source: BMC Biol. 2025 Aug 5;23:241. doi: 10.1186/s12915-025-02362-0 (PMC12326840; doi:10.1186/s12915-025-02362-0)
Supplement: Supplementary file 2 — Additional File 2: Tables S1–S6. Table S1 Summary statistics of syntenic blocks. Table S2 Summary of missing genes in vertebrate alignment for tetrapods. Table S3 Coverage of TADs within syntenic blocks across clades. Table S4 R2 and p values for TAD border comparison. Table S5 Syntenic blocks statistics across different α values. Table S6 Gene density in syntenic blocks with/without GRB. [file 12915_2025_2362_MOESM2_ESM.docx]

**SUPPLEMENTARY TABLES**

**Table S1** Summary statistics of syntenic blocks^§^

| **taxon** | **Minimum** | **Maximum** | **IQR** | **Mean** | **Median** | **std** | **Coverage** |
| --- | --- | --- | --- | --- | --- | --- | --- |
| **Rodents** | 2 | 1767 | 136.5 | 188.19 | 31 | 369.31 | 0.95 |
| **Cattle** | 3 | 1271 | 334 | 227.12 | 17 | 360.15 | 0.98 |
| **Primates** | 2 | 1517 | 275 | 214.82 | 47 | 341.37 | 0.97 |
| **Carnivores** | 2 | 1021 | 129 | 103.40 | 12 | 176.16 | 0.97 |
| **Ungulates** | 2 | 718 | 66.25 | 70.32 | 20 | 122.57 | 0.91 |
| **Laurasians** | 2 | 567 | 41 | 40.30 | 12.5 | 70.63 | 0.79 |
| **Glires** | 2 | 949 | 39.5 | 41.05 | 15 | 87.32 | 0.62 |
| **Eurarchontoglires** | 2 | 505 | 31.25 | 31.93 | 14 | 53.54 | 0.57 |
| **Mammals** | 2 | 245 | 17 | 19.13 | 8 | 29.27 | 0.43 |
| **Tetrapoda** | 2 | 125 | 8 | 9.27 | 5 | 11.38 | 0.26 |
| **Vertebrates** | 2 | 16 | 2 | 4.28 | 3 | 2.27 | 0.05 |

## ^§^This table provides a statistical overview of syntenic blocks for each taxonomic group alignment, detailing the minimum and maximum number of genes, interquartile range (IQR), median, standard deviation (std), and average coverage of syntenic genes for each genome.

**Table S2** Coverage of tads within syntenic blocks across clades^§^

| **taxon** | **Human** | **Rhesus** | **Mouse** | **Rat** | **Rabbit** | **Cat** | **Dog** | **Cow** | **Sheep** | **Pig** | **Chicken** | **Zebrafish** |
| --- | --- | --- | --- | --- | --- | --- | --- | --- | --- | --- | --- | --- |
| **Rodents** |  |  | 0.996 | 0.992 |  |  |  |  |  |  |  |  |
| **Cattle** |  |  |  |  |  |  |  | 1.0 | 1.0 |  |  |  |
| **Primates** | 0.997 | 0.999 |  |  |  |  |  |  |  |  |  |  |
| **Carnivores** |  |  |  |  |  | 0.949 | 0.946 |  |  |  |  |  |
| **Ungulates** |  |  |  |  |  |  |  | 0.991 | 0.99 | 0.999 |  |  |
| **Laurasians** |  |  |  |  |  | 0.845 | 0.856 | 0.852 | 0.854 | 0.85 |  |  |
| **Glires** |  |  | 0.815 | 0.811 | 0.887 |  |  |  |  |  |  |  |
| **Eurarchontoglires** | 0.781 | 0.765 | 0.76 | 0.758 | 0.827 |  |  |  |  |  |  |  |
| **Mammals** | 0.574 | 0.55 | 0.556 | 0.549 | 0.588 | 0.566 | 0.579 | 0.552 | 0.577 | 0.57 |  |  |
| **Tetrapoda** | 0.444 | 0.444 | 0.417 | 0.422 | 0.436 | 0.429 | 0.444 | 0.454 | 0.444 | 0.435 | 0.55 |  |
| **Vertebrates** | 0.129 | 0.116 | 0.122 | 0.125 | 0.119 | 0.124 | 0.128 | 0.114 | 0.131 | 0.127 | 0.185 | 0.267 |

^§^This table shows the proportion of TADs identified within syntenic blocks for each clade.

**Table S3** R² and P-values for TAD border comparison^§^

| **Taxon** | **R^2^** | **p-value** |
| --- | --- | --- |
| **Rodents** | 0.746111 | 9.9e-33 |
| **Cattle** | 0.805425 | 1.9e-07 |
| **Primates** | 0.917236 | 8.8e-42 |
| **Carnivores** | 0.901306 | 3.6e-122 |
| **Ungulates** | 0.799365 | 6.1e-242 |
| **Laurasians** | 0.737326 | 0.0e+00 |
| **Glires** | 0.652018 | 1.5e-146 |
| **Eurarchontoglires** | 0.668365 | 0.0e+00 |
| **Mammals** | 0.563330 | 0.0e+00 |
| **Tetrapoda** | 0.342046 | 0.0e+00 |

^§^This table shows the R² values and corresponding p-values for the comparison of TAD borders within syntenic blocks with the same number of TADs across the different clades.

**Table S4** Syntenic blocks statistics across different alpha values^§^

| **Alpha values** | **Number of blocks** | **Average number of genes per Mb** | **Average number of Tads per Mbp** |
| --- | --- | --- | --- |
| **0.01** | 36 | 18.49 | 6.2 |
| **0.09** | 20 | 7.7 | 1.63 |
| **0.17** | 13 | 7.74 | 1.48 |
| **0.26** | 8 | 8.91 | 1.84 |
| **0.34** | 18 | 7.27 | 1.8 |
| **0.67** | 46 | 9.35 | 2.41 |
| **1.0** | 19 | 7.47 | 1.72 |
| **2.0** | 12 | 8.53 | 1.78 |
| **3.6** | 88 | 15.06 | 4.07 |
| **5.2** | 131 | 21.41 | 6.5 |
| **6.8** | 29 | 12.98 | 2.66 |
| **8.4** | 59 | 21.21 | 6.44 |
| **10.0** | 6 | 41.2 | 12.07 |

^§^This table shows the genomic characteristics for different alpha values. The columns include the number of syntenic blocks, the average number of genes per megabase pair (Mbp), and the average number of TADs per megabase pair (Mbp) for each half-life group.
